# Optimized bilateral segmentation deep learning network for wild animal detection

**DOI:** 10.1371/journal.pone.0360422

**Published:** 2026-10-15

**Authors:** Srikanth Busa, Johnwesily Chappidi, Gunakala Archana, Anitha Rani Palakayala, Guggilam Venkata Vidya Lakshmi, Khasim Syed, Shaik Salma Asiya Begum

**Affiliations:** 1 School of Computer Science and Engineering, VIT- AP University, Guntur, Andhra Pradesh, India; 2 Department of CSE (AI&ML), Lakireddy Bali Reddy College of Engineering, Mylavaram, Andhra Pradesh, India; 3 Department of ACSE, Vignan’s Foundation for Science, Technology and Research, Guntur, Andhra Pradesh, India; 4 Department of Computer Science and Engineering, Koneru Lakshmaiah Education Foundation, Guntur, Andhra Pradesh, India; 5 School of Computer Science and Engineering, SRM University, Guntur, Andhra Pradesh, India; De Montfort University Faculty of Technology: De Montfort University Faculty of Computing Engineering and Media, UNITED KINGDOM OF GREAT BRITAIN AND NORTHERN IRELAND

## Abstract

The use of Deep learning (DL) for recognition of wild animals has become an important development in wildlife monitoring. The advances will include the use of multimodal data for improved accuracy and development of lightweight models for use in edge devices. As a result, DL will help contribute to global conservation efforts as it tackles the issues. The key novelty of the proposed framework is the integration of bilateral segmentation with DL to enhance relevant animal feature extraction while reducing the influence of complex background information. The paper proposes the Optimized Bilateral Segmentation DL Network that uses Harris Hawks Optimization (HHO) to improve Convolutional Neural Network (CNN) parameters for wild animal identification. The first step in the process was collecting datasets from various online sources. The image quality was improved through image processing methods such as Adaptive Histogram Equalization (AHE) as well as median filtering. The classifier was trained and validated using pre-processed images. This classifier combines the Bilateral Segmentation Deep Learning Network (BSDLN) with the HHO algorithm. CNN is utilized for detection and the weight parameter is utilized by HHO. In order to enhance model convergence also with performance, HHO dynamically adjusts the learning rate during training within the BSDLN. This balances speed and accuracy by adapting to gradients and losses behavior. By adaptively adjusting the learning rate, HHO and BSDLN optimize the CNN learning process and enhance convergence and classification performance. The suggested approach is put into practice in Python, and statistical metrics are used to assess its effectiveness. The results are compared with other approaches to verify the strategy’s efficacy. With just 0.2% FPR and 0.3% FNR, the suggested BSDLN performed the best overall, achieving 99.4% precision, 99.8% accuracy, 99.6% recall, 99.5% F1-score, and 99.3% specificity. The experimental findings show that the combination of HHO and bilateral segmentation enhances the efficacy of wild animal detection, offering a strong foundation for automated wildlife monitoring.

## Introduction

DL-based wild animal detection has become a game-changing tool for wildlife monitoring and protection. DL-based wild animal detection has become a game-changing tool for wildlife monitoring and protection. Wildlife migration observation has made use of animal monitoring and detecting techniques [1]. Finding and tracking migration patterns and assessing their impact on the ecosystem are the primary goals of animal behaviour study. Generally speaking, the constant stream of information and the cluttered backgrounds make it difficult for researchers to spot animal behaviour [2]. Rapid globalization is increasingly expected to need knowledge of and research on animal-vehicle collision fatalities [3]. The challenge of semantic annotation in the complex field of wildlife video has brought attention to the need for effective and trustworthy algorithms for tracking and detecting animals [4]. Thousands of animals are hurt or killed annually during agricultural mowing operations as a result of the wider operating widths and faster speeds of agricultural equipment [5]. These disputes have been more frequent in recent years. Therefore, this area has to be constantly watched to stop these creatures from entering or any other undesirable incursion [6]. Given that there is a greater chance of being hurt by lethal animals at night and in the morning, these problems are particularly significant [7]. Camera traps are installed in the majority of nature reserves to observe animal species and their behaviours [8]. Camera-trap systems use automatically triggered cameras to capture pictures or recordings of animals and their activities [9]. The use of remote thermal imaging cameras in animal research greatly lowers stress levels since the subject of the study does not need to be restrained or drugged [10]. More and more animals are being brought closer to populated areas, which might cause disputes and provide serious difficulties for farmers [11]. Numerous wild creatures are now homeless as a result of deforestation. They so assault people in search of food. Animals discovered in urban areas have become more common. Therefore, tracking them is crucial to ensuring human safety [12]. This frequently results in incidents that endanger human and animal lives. There are currently no effective mechanisms in place to stop these losses [13]. To give information for comprehending stereotyped animal actions, a number of automated detection techniques have been investigated [14]. More and more animals are being brought closer to populated areas, which might cause disputes and provide serious difficulties for farmers [15]. The animal has served as a sentinel to identify disease risk regions and to provide information about the pathogen’s evolutionary history [16]. Animal Home was created to address these issues by utilizing crowd sourcing and volunteer computing to swiftly analyze animal footage and to research computer vision-based automated video analysis methods [17]. Unmarked insect tracking is a challenging image-based tracking challenge. Earlier detection algorithms were created to study the behaviour of insects and tiny animals [18]. The project’s main goal is to create a system that will stop incidents like these and identify huge wild animals close to areas where humans and animals coexist in order to prevent deadly mishaps [19]. Its usage in additional surveillance domains is encouraged by the encouraging outcomes of the field test, which have led to a proposal for larger-scale implementation [20]. To enhance the performance of Wild animal detection, a novel OBSDLN is proposed int this study. To address the limitations of existing CNN-based wildlife detection and conventional segmentation approaches, an Optimized Bilateral Segmentation Deep Learning Network (OBSDLN) is proposed in this study. The novelty of the proposed framework lies in the task-specific integration of bilateral spatial-context segmentation, feature fusion, CNN-based animal identification, and Harris Hawks Optimization (HHO) within a unified wildlife detection pipeline. Unlike conventional CNN-based methods that directly perform classification from the input image, the proposed framework first separates relevant animal features from complex backgrounds through the Spatial Path (SP) and Context Path (CP). The Feature Fusion Module subsequently combines spatial details and contextual information to obtain more discriminative representations for animal identification. In addition, HHO is incorporated to optimize the CNN parameters, thereby improving the learning and detection performance of the network. Thus, the proposed OBSDLN is designed to jointly address background complexity, feature representation, and parameter optimization rather than relying solely on conventional CNN-based detection.

### Contributions

The main contributions are as follows:

1. A bilateral segmentation architecture combining a Spatial Path and Context Path to jointly preserve spatial details and capture high-level contextual information for wildlife images with complex backgrounds.
2. A Feature Fusion Module that integrates spatial and contextual representations before animal identification.
3. Integration of Harris Hawks Optimization with the CNN-based identification stage for systematic parameter optimization.
4. An integrated preprocessing–segmentation–identification framework for five-class wildlife identification covering 90 animal species.

The following section gives information about the relevant works, while the Proposed System Section provides the details of the proposed methodology and also the dataset description. The performance analysis of the proposed method is explained in Results and Discussion Section, besides discussing the evaluation parameters. The Conclusion Section provides a conclusion, finally.

## Literature review

Munian, Y. et al. [3] proposed an intelligent wildlife detection system that uses thermographic images, CNN, and HOG to detect animals near roads at night and improve vehicle safety. The proposed method was benchmarked against several CNN and machine learning algorithms, including VGG16, SVM, RF, DT, LR, and GNB. However, its performance decrease in adverse weather conditions, complex backgrounds, and with limited thermographic training data. Magaldi H. et al. [4] developed a DL -based approach to automate wildlife identification from camera-trap images for monitoring biodiversity in tropical forests. The method reduces manual analysis effort and supports wildlife research and conservation by accurately identifying target species. However, it requires large annotated datasets, high computational resources, and its performance vary across different forest environments and species. Xie P. et al. [5] have proposed a weakly supervised approach that generates bounding boxes for large camera-trap datasets using a small set of manually labeled images. The method trains an initial animal detector, generates bounding boxes automatically, and improves detection performance in terms of mean Average Precision (mAP). However, its performance based on the accuracy of the initial detector, and incorrect bounding boxes may reduce overall detection accuracy. Norouzzadeh, M.S. et al. [21] suggested a DL-based approach to automatically extract information from motion-sensor camera-trap images for wildlife monitoring. The method enables efficient and cost-effective collection of animal location and behavior data, supporting biodiversity research and conservation. However, it requires large labeled datasets, high computational resources, and its performance reduced under occlusions, rare species, and complex environments. Vélez, J. et al. [22] presented an AI-based approach using DL and CNNs to automatically detect and classify objects like humans, animals, as well as vehicles from camera-trap images. The method reduces the manual effort required to process large-scale wildlife monitoring data. However, it requires extensive labeled training data, high computational resources, and performance decline under varying environmental conditions. Dave, B. et al.[23], have introduced the YOLOv8 architecture, which can identify four different species: bears, tigers, leopards, and lions. The dataset was created using pre-existing Kaggle datasets, YouTube videos, and a variety of documentaries. 1619 photos with annotations for four different item categories make up this collection. Several forms of augmentation were used to the dataset photos in order to increase our model’s accuracy. With a mAP of 94.3%, the trained extra-large model proved to be successful in real-time wild animal detection at 20 FPS. Peng, J. et al.[24], have created a number of automated and semi-automated methods to recognize wild animals in UAS photos. The microscopic recognition performance of the animal patch set was enhanced by characteristic cycle reduction and anchored length tuning. The hard negative class raised the F1-score from 0.44 to 0.86, indicating a substantial reduction in false positives throughout the whole UAS picture set. Zhang. T. et al.[25], have demonstrated how to handle large amounts of data from field security cameras using CNN-based detectors. In an omni-supervised learning environment, several species in the wild, including kangaroos, emus, dingos, birds, and wildcats were identified. The unlabelled security camera data was filtered using a data distillation technique and used for training. A substantial file of complete high definition (HD) wild animal surveillance photos was also created to assess the viability using information gathered from many national parks in Queensland of Australia. This dataset was also made publicly accessible. Numerous tests demonstrate that intelligent use of unlabelled data can significantly enhance the detection and pose estimation outcomes. The existing works demonstrate strong performance under specific acquisition conditions; however, most rely either on direct CNN-based classification, computationally intensive detection architectures, or large annotated datasets. In addition, approaches designed for adverse environments may require specialized modalities such as thermal or aerial imagery. These limitations motivate a framework that explicitly separates spatial and contextual information before identification while incorporating parameter optimization. The literature review summary is displayed in Table 1.

**Table 1:** Summary of literature review.

Ref	Methods	Dataset	Advantages	Limitations
Munian et al. [3]	Thermographic CNN-HOG wildlife detection	Nighttime thermographic wildlife image dataset	Improves nighttime animal detection accuracy	Weather affects detection performance
Magaldi *et al.* [4]	DL wildlife species identification	Tropical forest camera-trap image dataset	Supports automated biodiversity monitoring	Needs annotated data
Xie et al. [5]	Weakly supervised bounding-box generation method	GSBBC	Reduces manual annotation effort	Initial detector errors reduce accuracy
Norouzzadeh *et al.* [21]	DL wildlife information extraction	Motion-sensor camera-trap wildlife images	Automated monitoring, improved accuracy	Needs large datasets
vélez *et al.* [22]	CNN-based wildlife object classification	Large-scale camera-trap image dataset	Reduces manual image processing effort	Requires labeled data
Dave, B. et al. [23]	YOLOv8-X with data augmentation	1,619 annotated images of bears, tigers, leopards and lions from Kaggle, YouTube and documentaries	94.3% mAP; 20 FPS; suitable for real-time detection	Only four species; relatively small and heterogeneous dataset; limited generalization
Peng, J. et al. [24]	Automated/semi-automated UAS recognition; characteristic cycle reduction, anchor-length tuning and hard-negative learning	UAS/aerial wildlife images and animal patch dataset	Improved small-animal recognition; F1-score increased from 0.44 to 0.86; reduced false positives	Small animals in aerial images are difficult to detect; mainly applicable to UAS imagery
Zhang, T. et al. [25]	CNN detectors with omni-supervised learning and data distillation	HD wildlife surveillance images from Queensland national parks; kangaroos, emus, dingos, birds and wildcats	Efficient use of unlabeled data; improved detection and pose estimation; public dataset	Requires large unlabeled datasets; computationally demanding; dependent on surveillance-image quality

### Research gaps

Many of the wildlife monitoring techniques based on DL technology have emerged, but multiple problems persist. One of the weaknesses is connected with the fact that DL strategies for wildlife monitoring can be implemented only in the presence of large labeled datasets and sophisticated technologies capable of performing complex calculations [21]. The performance of animal detection strategies based on AI relies on big labeled datasets while being influenced by various environmental factors [22]. The CNN model of thermographic detection exhibits a low level of capability in adverse weather conditions and requires a lot of thermography datasets for training [23]. The Deep Forest Vision-based wildlife identification approach demands large annotated datasets and its performance varies across different forest environments and species [24]. The weakly supervised bounding-box generation method relies heavily on the accuracy of the initial detector, where incorrect bounding boxes reduce overall detection performance [25]. Therefore, an Optimized Bilateral Segmentation Deep Learning Network (OBSDLN) integrated with HHO is proposed to improve detection accuracy, robustness, and computational efficiency for wildlife detection.

## Proposed system

Fig 1 depicts the complete architecture of the proposed method. The suggested approach employs four distinct phases: data collection, segmentation, data pre-processing, and Wild animal identification. The images are preprocessed using a median filter and AHE technique is applied to improve the quality of images. BSDLN is then used to train these pre-processed images. Finally, with the HHO algorithm for wild animal detection. Although multiple diagnosing techniques in determining the detection have been established, still it is essential to put forth huge effort in accurate detection.

**Fig 1:**
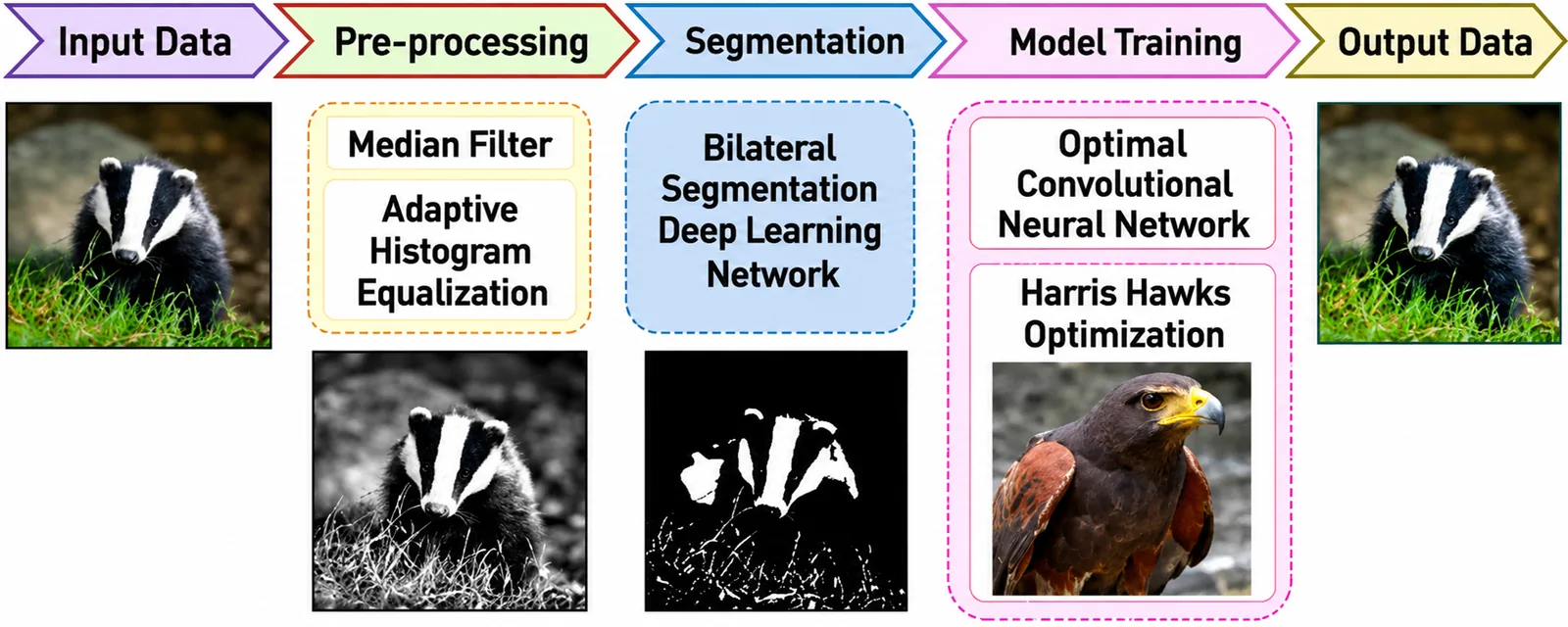
Architecture of the proposed system.

### Dataset description

This manuscript reports studies which does not involve animals or tissues. The data set utilized in this study was The Animal Image Dataset–90 Distinct Animals, which is a benchmark dataset that can be easily accessed for wildlife classification and detection studies [26]. It can be accessed through https://www.kaggle.com/datasets/iamsouravbanerjee/animal-image-dataset-90-different-animals. The dataset comprises approximately 5400 RGB images of 90 animal species, with each class containing about 60 images and these are organized into five higher-level classes for the classification task. The images are sourced from the internet and feature a variety of conditions in regard to pose, background, lighting, scope, and view angles, making the dataset suitable for testing efficiency of DL models. The dataset was divided into 80% training, 10% validation, and 10% testing subsets, corresponding to approximately 4320, 540, and 540 images, respectively, with approximately 48 training, 6 validation, and 6 testing images per species. Dataset has a balanced number of training as well as testing images that can guarantee reliable training of any model. As the dataset is well balanced among the classes, data augmentation techniques were not utilized in this study.

### Image preprocessing

In wildlife animal detection, pre-processing is essential to make sure that the data is properly organized, cleaned, and improved for useful analysis. A median filter and AHE are used in this study to reduce noise and enhance image contrast respectively.

### Median filter

It is a non-stationary method that preserves visual details while lowering or filtering noise in a picture. By creating values as medians, values on photographs will be changed or substituted. The median has been stored in place of the noise value when the grey level is used. The grey level is represented by the average, the highest by the max, and the lowest value by the min. *p (x, y)* is the center of manipulation of the median filter [27].


$$\begin{array}{c} p(x,y)=median\{m[i,j],(i,j)\in w\} \end{array}$$ (1)

Where p(x,y) is a matrix of results labelled p with x,y is arrow multiplied by a column. {m[i,j],(i,j)∈w} represents a matrix quantity of the processed image or the arranged related elements. When the neighbour’s pixel value is detected by the median filter, it will match the value that the neighbour’s pixel value represents. A count of nearby neighbours will be used to replace the pixel value at the midpoint value. When the median value is even on the neighbouring pixel, it will be used with the mean equal to both middle values. After noise reduction with the median filter, the picture is enhanced using AHE.

### Adaptive histogram equalization

It is a method of processing digital pictures that improves contrast in images. In contrast to conventional histogram equalization, the adaptive approach improves the contrast locally. The picture is separated into many segments, and the equalization of the histogram for each block is then determined [28].

### BSDLN for Segmentation

The Wildlife animal detection is the process of identifying and recognizing animals in their natural habitats using a range of technical tools as well as methods. The aim of wildlife animal detection is to follow and observe animal populations in their natural habitats. This section begins by providing a detailed example of Bilateral Segmentation Network using CP as well as Spatial Path (SP). Further, the effectiveness of these two routes is explained independently. Finally, the usage of Feature Fusion Module and the BSDLN complete architecture to integrate the features of these two paths.

#### Spatial path.

THE pyramid dumping modules, atrous geographical triangle pooling, or large kernel are some of the methods used in some of the current semantic segmentation algorithms in an effort to capture a broad enough receptive field. While others use dilated convolution to maintain the resolution of the image being entered in order to encode adequate spatial information. These techniques show that attaining great accuracy requires both the receptive field and spatial information. Meeting these two objectives at the same time is challenging. In particular, current latest techniques make use of lightweight base models or minimal input images to accelerate real-time semantic segmentation [29]. While the lightweight model uses channel pruning to erase spatial information, the input image’s small size eliminates much of the spatial data in the original picture. In light of this finding, suggest an sp that encodes rich spatial information while maintaining the original input image’s spatial dimensions. There are three layers in the sp. Each layer has a stride of 2 convolutions, batch normalization, as well as Relu. As a result, 1/8thpart of the original image’s output feature maps is extracted via this approach. Feature maps may encode a lot of data about space because of their large spatial scale. The structure’s specifics are shown in Fig 2.

**Fig 2:**
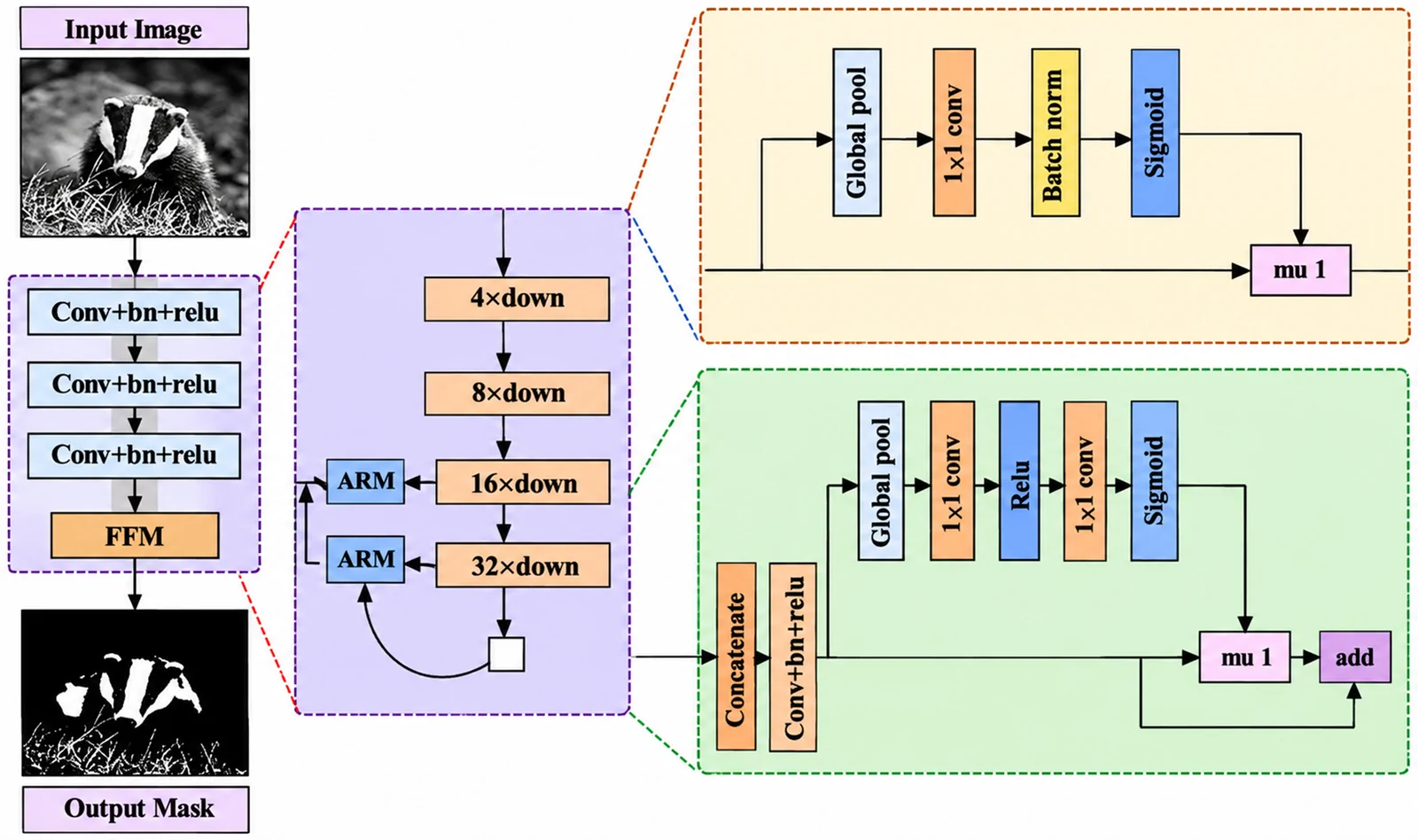
Structure of BSDLN.

#### Context path.

The context-based path seeks to provide an adequate receptive field, but the SP retains rich spatial data. The receptive field has a significant impact on how well the semantic segmenting job is performed. The pyramidal pooling section atrous spatial pyramid pooling, sometimes referred to as the “large kernel,” has been employed in some techniques to expand the field of perception. However, the low pace is caused by the computer and memory demands of these processes. The CP that takes into account both the enormous receptive field and effective computation simultaneously, is suggested. The contextual path provides a wide field for reception by using the worldwide average pooling and an extremely light model. High level semantic context information is encoded in the vast receptive field obtained by the lightweight model, for instance, Xception in this work. The compact model combines the qualities of the last two phases using the U-shape structure. It is an unfinished U-shaped design.

#### Network architecture.

For instantaneous semantic segmentation using the SP and the CP, a bilateral segmentation network is suggested, as illustrated in Fig 2. Use three convolutional layers with the trained beforehand Xception, among as the model for the path’s surroundings and stride as the SP. The final forecast is then created by fusing the output features of these two routes. It can simultaneously achieve high accuracy and real-time performance. Initially, concentrate on the element of practical calculation [30]. The SP only has three convolution layers, despite its huge spatial extent. It is not computationally demanding as a result. Quickly down-sample the CP using a lightweight model. Additionally, the efficiency is much increased because these two pathways compute simultaneously. Second, talk about this network’s accuracy. For improved performance, them work in random with one another.

***Module for feature fusion:*** The two pathways’ features differ in terms of feature representation level. As a result, unable to simply summarize these characteristics. Rich detail information is primarily encoded in the spatial data that the SP records. Furthermore, the Context Path’s output feature primarily encapsulates context data. Stated differently, Context Path’s output feature is high level, whereas SP is low level. To fuse these features, thus suggest a particular Feature Fusion Module. To address the varying levels of features, begin by merging the output features from both the SP as well as the CP. Then, batch normalization is applied to ensure the feature scales are balanced. Following this, the combined feature is pooled into a feature vector, and a weight vector is computed similarly to Segmentation Network. Feature selection and integration are made possible by this weight vector’s assistance in re-weighting the features (Fig 3).

**Fig 3:**
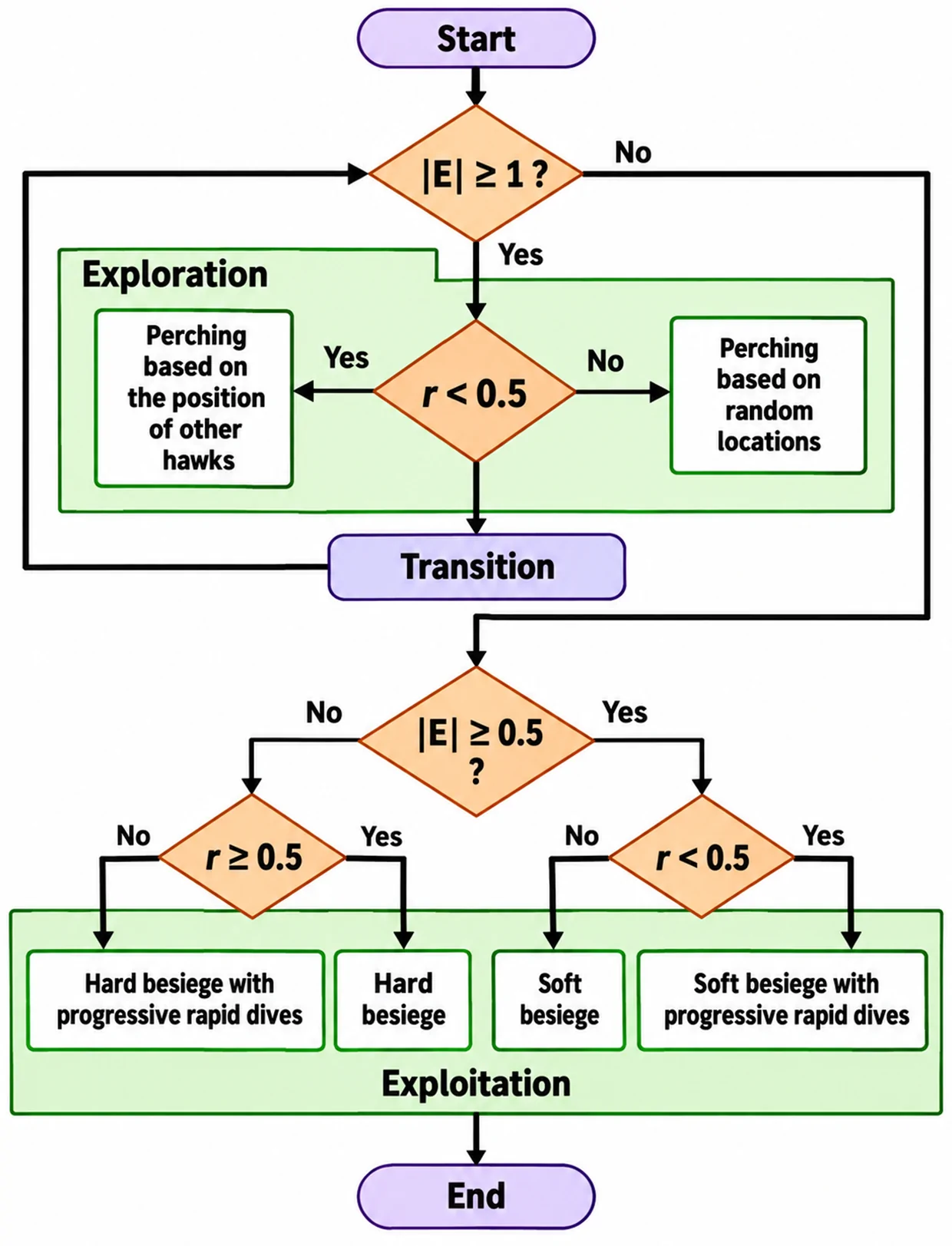
Flow chart of HHO.

***Attention refinement Module (ARM):*** Let’s add an ARM to the CP to improve the features at every turn. The ARM creates a feature-directing concentration vector learning and extracts global context via global average pooling, as seen in Fig 4. With this method, Context Path’s output characteristics in each level is improved, and global context information is easily included without the need for upsampling. As a result, it does so with little computational values.

**Fig 4:**
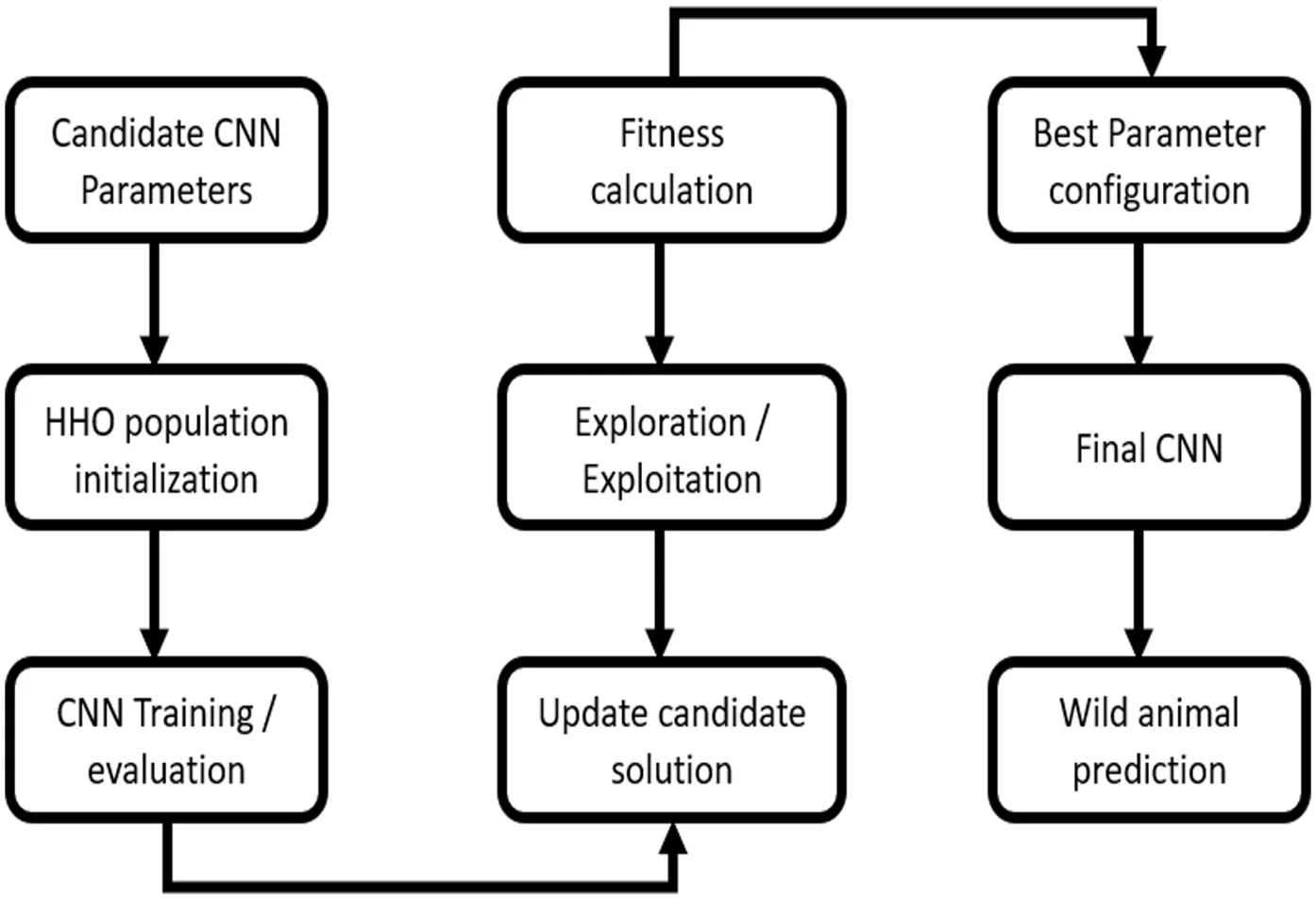
Flow chart of proposed method using HHO.

### Optimal convolutional neural network (OCNN)

Typically, CNN is composed of numerous layers that can be combined based on their functionalities.

Numerous computational tasks can be linked to Convolutional Neural Networks. To identify the wild animal, the system consists of an input layer, three Convolutional as well as max pooling layers, as well as a soft max fully connected output layer. CNN has proven to be successful in every application involving image processing. The proposed system handles the output in multiple layers after introducing the input image to the underlying transformation layer. The converter layer’s output is contributed to the maximum pooling layer, which eliminates unwanted pixels. CNN recognizes the wild animal and shrinks its size without sacrificing its characteristics. The clear explanation of each layer is described below.

#### Convolutional layer.

The convolutional layer is the primary layer of the CNN network. In this case, a convolution filter is used to apply the convolution to the input data in order to produce a feature map. It consists of a convolution mask, bias terms, and a function value. Together, these make output of the layer [31]. In terms of mathematics, if ${\;w}_{ij}^{m}$ and $b_{i}^{m}$ indicate the weights as well as biases of the $i^{th}$ filter of the $m^{th}$ convolutional layer and $A_{i}^{m}$ represent its mapping of activation, Where, ⨂  is the operator for convolution.


$$\begin{array}{c} A_{i}^{m}=\sum_{j\in F_{i}}A_{j}^{m-\text{1}}\;⨂{\;w}_{ij}^{m}+b_{i}^{m} \end{array}$$ (2)

#### Max pooling layer.

The convolutional layer is followed by the max pooling layer. It has the same number of planes as the convolutional layer. this layer’s goal is to make the feature map smaller. It divides the image into 2x2 squares and averages them. The pooling layer protects the relative data between features rather than the particular link. When feature map a is sent to maximum pooling layer, maxi function is used for feature map A, which gives a pooled feature map P as yield,


$$\begin{array}{c} P_{i}={Max}_{j\epsilon R_{j}}A_{i} \end{array}$$ (3)

#### Output layer.

The fully connected layer receives the flattened output from the final pooling or convolutional layer. every neuron in the fully connected layer is connected to every other neuron in the previous layer. The neural system’s yield is converted into likelihood for each class using a soft max work. The HHO is applied to the CNN way to accurately adjust its parameters, improving the outcome. The method that has been presented makes use of a CNN, to detect the wild animal. the hyper parameter value does not considerably improve the performance of the conventional CNN. Consequently, the hyper parameter is tuned using the HHO algorithm. Below is a detailed description of the HHO algorithm. The detailed architectural configuration of the proposed BSDLN can be observed from Table 2 and the detailed architecture of the CNN-based animal identification network can be observed from Table 3.

**Table 2:** The detailed architectural configuration of the proposed BSDLN.

Module	Layer	Kernel/ Operation	Filters/ Output Channels	Stride	Activation	Output/ Function
Input	Input image	—	3	—	—	Preprocessed RGB image
Spatial Path	SP-Conv1	Convolution	3	2	ReLU	1/2 spatial resolution
	Batch Normalization	Batch Normalization				Normalization
	SP-Conv2	Convolution	3	2	ReLU	1/4 spatial resolution
	Batch Normalization	Batch Normalization				Normalization
	SP-Conv3	Convolution	3	2	ReLU	1/8 spatial resolution
Context Path	Xception	Pre-trained Xception			–	High-level contextual features
	Global Average Pooling	GAP				Global context vector
	ARM	Attention Refinement				Refined contextual features
Feature Fusion	Concatenation	SP + CP	256	2	ReLU	Combined feature representation
	Batch Normalization	Batch Normalization				Balanced feature scales
	Pooling	Global Average Pooling				Feature vector
	Weight vector	Feature re-weighting				Reweighted fused features
Output	Prediction	Fusion/output layer		—	—	Segmentation output

**Table 3:** The detailed architecture of the CNN-based animal identification network.

Layer	Input Size	Kernel	Filters/ Units	Stride	Output Size	Parameters
Input	227 × 227 × 3	—	3	—	227 × 227 × 3	0
Conv1	227 × 227 × 3	3 × 3	32	1	114 × 114 × 32	896
MaxPool1	114 × 114 × 32	2 × 2	—	2	57 × 57 × 32	0
Conv2	57 × 57 × 32	3 × 3	64	1	29 × 29 × 64	18,496
MaxPool2	29 × 29 × 64	2 × 2	—	2	15 × 15 × 64	0
Conv3	15 × 15 × 64	3 × 3	128	1	8 × 8 × 128	73,856
MaxPool3	8 × 8 × 128	2 × 2	—	2	4 × 4 × 128	0
Flatten	4 × 4 × 128	—	—	—	2048	0
Fully Connected	2048	—	5	—	5	10,245
Softmax	5	—	5	—	5	0

### Harris Hawks Optimization (HHO) Algorithm

A novel bio inspired method called HHO [32] was developed in response to some Harris Hawks working together to seek prey. Typically, rabbits that are adept at evading a Harris Hawk attack are the primary target of the hawk [31]. As a result, Harris Hawks try to launch a coordinated surprise attack to sap the bunnies’ vitality. The basic structure of the HHO algorithm is based on this concept. Similar to other bio-inspired algorithms, the HHO comprises two steps, which are described below.

### Exploration level

Harris Hawks are frequently positioned in higher trees to get a wider view of their prey. This procedure might be regarded as the Harris Hawk’s exploratory stage. This procedure’s mathematical model is provided below:


$$\begin{array}{c} H_{i+\text{1}}=\left\{ \begin{array}{c} @lrH_{rand}-r_{I}*\left|H_{rand}-H\right|h\;\geq \text{0}.\text{5}\\ H_{rabbit}-\overset{―}{H}-r_{\text{3}}*\left(a+r_{\text{4}}*(b-a)\right)h\;<\text{0}.\text{5} \end{array}\right. \end{array}$$ (4)

Here, If h<0.5, sit near their prey, which is a rabbit, and if h≥0.5, those who sit atop any talltree between 0 and 1. $H_{i}\;and\;H_{i+\text{1}}$ explains the Harris Hawk’s present location as well as its future location.$\;H_{rand}$, $H_{rabbit}$ AND $\overset{―}{H}$ describes the Harris Hawk’s average position, the rabbit’s position, and the random position respectively, a and b represent the position’s upper and lower bounds while i signifies the current iteration, as well as $r_{\text{1}}$-$r_{\text{4}}\;$ include arbitrary numbers between 0 and 1.

### The meeting point of exploration and exploitation

An important component of creating an interface between the degrees of exploration and exploitation is the energy lost by the rabbits during their escape. The following equation is utilized to formulate this step.


$$\begin{array}{c} E=\text{2}E_{\text{0}}\left(\text{1}-\frac{t}{T}\right) \end{array}$$ (5)

where, T represents the most iterations possible, t represents the current iteration, $E_{\text{0}}$ represents the beginning energy that ranges randomly from −1 and 1, and E denotes the rabbit’s the breakdown of energy. Using a random number between −2 and 2, which falls as the number of iterations rises. According to this debate, as soon as the rabbit’s energy loss exceeds one, i.e., |𝐸| ≥ 1, during the exploring stage, will try to find an avoiding predator and if |𝐸| < 1, the exhausted victim will thereafter be hunted by the Harris Hawks (exploitation stage).

### Exploitation level

While hunting, the Harris Hawks startle the rabbits by attacking them. This process is determined by the attitude of the attacked the amount of running energy left in the rabbits. This residual energy is calculated using a random number, r, in the interval [0, 1]. A successful escape is indicated if ‘r’ is less than 0.5; an unsuccessful one is indicated if ‘r’ is more than or equal to 0.5. Based on the previously gathered data, Hunting (exploitation) levels are taken into account in four separate states, as indicated below.

***Soft besiege:*** The Harris Hawks surround the victim as it gradually loses energy before attacking it abruptly since it still has enough energy to escape. The following is a model for this:


$$\begin{array}{c} H_{t+\text{1}}=H_{rabbit}-H_{t}-E\times |\alpha \times H_{rabbit}-H_{t}| \end{array}$$ (6)

where $\alpha =\text{2}-\text{2}r_{\text{5}},r_{\text{5}}\;\epsilon \;[\text{0},\text{1}].\;H_{t+\text{1}}$ represents the updated position of the Harris Hawk representing the new solution generated in the next iteration, $H_{rabbit}$ denotes the position of the rabbit representing the current best solution identified during the search process, $H_{t}$ Indicates the current position of the Harris Hawk before moving toward the rabbit’s current best position, E represents rabbit’s escaping energy controlling hawk movement, gradually decreasing as iterations progress from exploration to exploitation.

***Hard besiege:*** Assumptions |E|<0.5  also with r ≥0.5  indicates when the victim is tiered as well as has used all of its energy. The Harris Hawks encircle it at this period and launch a forceful surprise assault on their target.


$$\begin{array}{c} H_{t+\text{1}}=H_{rabbit}-E\times \left|H_{rabbit}-H_{t}\right| \end{array}$$ (7)

***Soft besiege with progressive rapid dives:*** Assumptions |E| < 0.5 and r >= 0.5 indicate when the prey is tiered and has used all of its energy. The Harris Hawks encircle it at this period and launch a forceful surprise assault on their target:


$$\begin{array}{c} I=H_{rabbit}-E\times |\alpha \times H_{rabbit}-H_{t}| \end{array}$$ (8)

I denotes intermediate position of the harris hawk calculated before performing a rapid dive toward the rabbit. The decision for Harris Hawks to dive depends on the movement of their prey. When the prey attempts to escape by jumping, the hawks swiftly dive to using the LF method as explained below, catch the escaping bunnies:


$$\begin{array}{c} J=I+S\times LF(D) \end{array}$$ (9)

where J  represents the final updated position of the Harris Hawk after performing a rapid dive toward the escaping rabbit, S represents the random step vector introducing unpredictable movement during the hawk’s rapid dive toward the escaping prey, LF(D) represents the levy flight function generating the long and short random movements to imitate the hawk’s rapid dive. Where random integers between u and v are represented by 0 and 1, β is a fixed integer equal to 1.5, and the following is how the last next place is attained:


$$\begin{array}{c} {\sigma =\left(\frac{\Gamma (\beta +\text{1})\times \text{sin}\left(\frac{\pi \times \beta }{\text{2}}\right)}{\Gamma \left(\frac{\beta +\text{1}}{\text{2}}\right)\times \beta \times \text{2}\left(\frac{\beta -\text{1}}{\text{2}}\right)}\right)}^{\frac{\text{1}}{\beta }} \end{array}$$ (10)

$$\begin{array}{c} H_{t+\text{1}}=\left\{ \begin{array}{c} @lrI,\;F(I)<F(H_{t})\\ J,F(I)<F(H_{t}) \end{array}\right. \end{array}$$ (11)

where, F(I), F(j) and $F\left(H_{t}\right)$ are the values of the purpose of fitness stand at I, J and $H_{t}$ position.

***Hard besiege with progressive rapid dives:*** Assumptions |E| < 0.5 as well as r < 0.5 signifies that the prey is exhausted, as well as barely encircle it before launching an unexpected attack to pursue it. This condition is identical to the previous one, except instead of using the current position (), Harris Hawke’s average position () is utilized. Using the prey energy, the HHO algorithm changes the Harris Hawks’ location until the most iterations are reached is achieved. The BSDLN parameter was optimized effectively through the use of the HHO algorithm.

***Role of HHO in CNN Parameter Optimization:*** In the proposed framework, Harris Hawks Optimization (HHO) is employed as a metaheuristic optimization mechanism for optimizing the parameters of the CNN-based animal identification stage. Each candidate solution in the HHO population represents a set of candidate CNN parameter values. The quality of each candidate solution is evaluated using the CNN objective/fitness function on the training data. During the exploration stage, HHO searches different regions of the parameter space, whereas during the exploitation stage, the search is progressively concentrated around the best-performing candidate solution. The rabbit position in the HHO formulation represents the current best solution, while the hawk positions represent candidate parameter configurations. The fitness value is used to determine whether a candidate configuration improves the CNN detection performance. The optimized parameter configuration is subsequently used for the final CNN-based wild-animal identification. Thus, HHO is not used as an additional feature-extraction layer; rather, it acts as an optimization mechanism for the CNN parameter search.

### Hyper parameters

Table 4 depicts the parameter configuration of the suggested method. The parameter settings table summarizes the key configurations used to train the proposed model. It outlines choices for optimization, learning rate, batch size, epochs, loss functions, activations, dropout, and weight initialization. These settings ensure stable convergence, effective segmentation and classification, and improved generalization performance.

**Table 4:** Hyperparameters configuration of OBSDLN.

Parameter	Value
Optimizer	Adam
Initial Learning rate	0.001
Loss Function (Segmentation)	Dice Loss + Cross Entropy
Epochs	100
Batch size	32
Activation functions	Rectified Linear Unit (ReLU), Softmax
Dropout Rate	0.3
Weight Initialization	He Normal

## Results and discussion

This section includes comparisons, performance metrics, and a description of the dataset. The computational framework was implemented in a Python environment of version 3.12.12, utilizing the infrastructure of Google Colab. For manipulating the data and performing various operations in the process of feature development, the Pandas and NumPy libraries were used. Also, the scikit-learn library was utilized as a foundation for stratified data partitioning.

### Ablation study

The Ablation study (Fig 17 and Table 5) demonstrates that the proposed classification framework benefits from the cumulative integration of data preprocessing, segmentation, CNN-based feature extraction, and HHO-based optimization. Preprocessing improves the quality of the input representation, while the CNN provides substantial gains in discriminative feature learning. Although segmentation alone results in a marginal performance decrease, its integration with CNN-based feature extraction leads to a substantial improvement, indicating that the segmented representations are more effectively exploited by the deep learning model. Finally, the incorporation of HHO optimization further improves the classification performance, achieving 0.99 accuracy, precision, recall, and F1-score. These results substantiate the effectiveness of the proposed integrated framework and demonstrate the complementary contribution of its major components (Tables 6 and 7).

**Table 5:** Ablation study analysis.

Configuration	Acc	Prec	Rec	F1-Sc
Original Data	0.93	0.92	0.91	0.91
Preprocessed Data	0.96	0.95	0.95	0.95
Preprocessed and Segmented Data	0.95	0.94	0.94	0.94
Preprocessed and Segmented Data with CNN	0.98	0.98	0.97	0.97
Preprocessed and Segmented Data with CNN and HHO optimization (Proposed Model)	0.99	0.99	0.99	0.99

*Acc-Accuracy; Prec-Precision; Rec-Recall; F1-Sc: F1-score.

**Table 6:** Comparison analysis.

Method	Acc	Prec	Rec	F1-Sc	Spec	FPR	FNR
**Proposed**	0.998	0.994	0.996	0.995	0.993	0.002	0.003
YOLOv8	0.955	0.990	0.970	0.968	0.945	0.114	0.046
DenseNet	0.985	0.987	0.971	0.979	0.990	0.004	0.028
ResNet	0.967	0.947	0.937	0.942	0.939	0.098	0.062
CNN	0.965	0.919	0.933	0.926	0.926	0.125	0.066
DNN	0.948	0.901	0.902	0.902	0.896	0.157	0.097

*Acc-Accuracy; Prec-Precision; Rec-Recall; Spec-Specificity; F1-Sc: F1-score; FPR-False positive rate; FNR-False negative rate.

**Table 7:** State-of-the-art works comparison.

Source	Methods	Accuracy
Christiansen, P. et al. [5]	k-NN	93.5%
Dave, B et al. [23]	Yolov8	94.3%
Peng, J.et al [24].	R-CNN	96.8%
Chandrakar, R. et al. [7]	CNN	98.2%
**Proposed (OBSDLN)**	**BSDLN**	**99%**

### Evaluation parameters

The evaluation Parameters are deployed in the developed suggested method, which evaluates the performance based on Wild animal detection. The employed evaluation parameters: are specificity, precision, recall, accuracy, F1-Score, FNR and FPR.

#### Specificity.

The Specificity measure is determined as the proportion of the number of formed true negatives for the positive and negative classes. The below-mentioned equation is deployed to calculate the Specificity measure.


$$\begin{array}{c} \text{Specificity}=\frac{{\text{TRE}}_{\text{NEG}}}{{\text{TRE}}_{\text{NEG}}+{\text{FSE}}_{\text{PSV}}} \end{array}$$ (12)

#### Precision.

The precision parameter is employed for measuring the accurate positive results in detection, this measure utilizes the sum of positive results that are expected for dividing on this.


$$\begin{array}{c} \text{Precision}=\frac{{\text{TRE}}_{\text{PSV}}}{{\text{TRE}}_{\text{PSV}}+{\text{FSE}}_{\text{PSV}}} \end{array}$$ (13)

#### Accuracy.

For each result, the ratio of the correct detection is stated as the accuracy parameter and it is deployed in measuring the proposed model and how it performs detection. The computation of the accuracy parameter is denoted in the below equation.


$$\begin{array}{c} \text{Accuracy}=\frac{{\text{TRE}}_{\text{PSV}}\;+{\text{TRE}}_{\text{NEG}}}{{\text{TRE}}_{\text{PSV}}+\;{\text{TRE}}_{\text{NEG}}+{\text{FSE}}_{\text{PSV}}+\;{\text{FSE}}_{\text{NEG}}} \end{array}$$ (14)

#### F1-score.

F1-score is the loaded a typical basis, or cohesive median, of both recall and precision. This offers a comprehensive assessment of a method effectiveness regarding precise class predictions.


$$\begin{array}{c} \text{F1}-\text{Score}=\frac{{\text{2T}}_{\text{r}}}{{\text{2T}}_{\text{r}}+{\text{F}}_{\text{r}}+{\text{F}}_{\text{s}}} \end{array}$$ (15)

#### Recall.

In tasks involving categorization and information retrieval, recall is a frequently used assessment metric. It assesses a model’s accuracy in locating every pertinent instance in the dataset. The sensitivity or true positive rate are other names for recall.


$$\begin{array}{c} Recall=\frac{{TRE}_{PSV}}{{TRE}_{PSV}\;+\;{FSE}_{NEG}} \end{array}$$ (16)

#### False Positive Rate (FPR).

One statistic for assessing a classification model’s performance is the FPR. It assesses the percentage of the bad circumstances that are mistakenly categorized as good.


$$\begin{array}{c} \text{FPR}=\frac{{\text{FSE}}_{\text{PSV}}}{{\text{FSE}}_{\text{PSV}}\;+\;{\text{TRE}}_{\text{NEG}}} \end{array}$$ (17)

#### False Negative Rate (FNR).

The percentage of positive examples that are mistakenly labelled as harmful in classification tasks is known as the FNR. It displays how frequently positive instances are missed by a model.


$$\begin{array}{c} \text{FNR}=\frac{{\text{FSE}}_{\text{NEG}}}{{\text{FSE}}_{\text{NEG}}\;+\;{\text{TRE}}_{\text{PSV}}} \end{array}$$ (18)

### Performance metrics

The performance of the proposed method was contrasted with existing algorithms such as DenseNet, ResNet, CNN, and DNN based on different metrics. The results of the comparison can be found in Fig 5–Fig 11.

**Fig 5:**
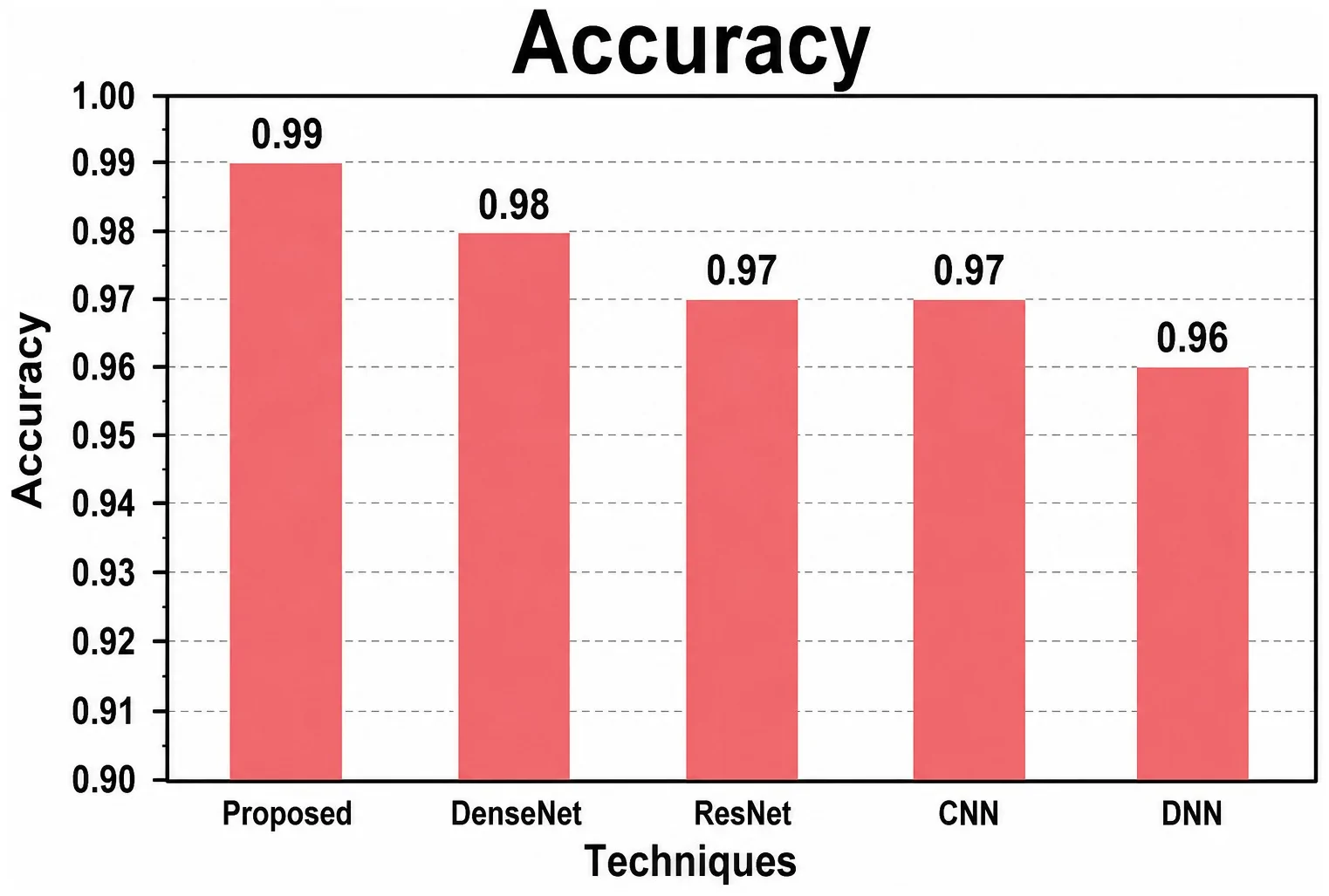
Accuracy.

**Fig 6:**
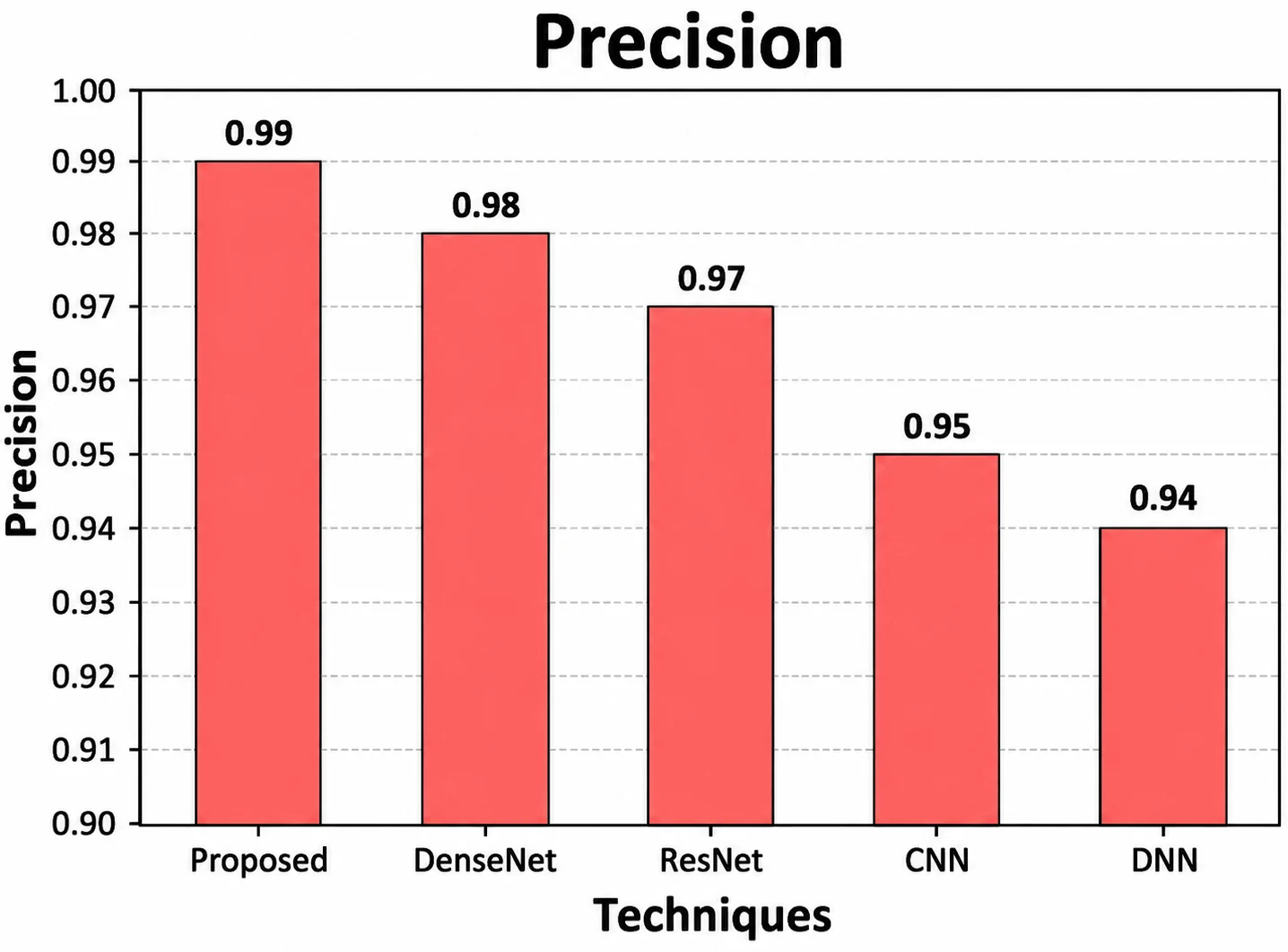
Precision.

**Fig 7:**
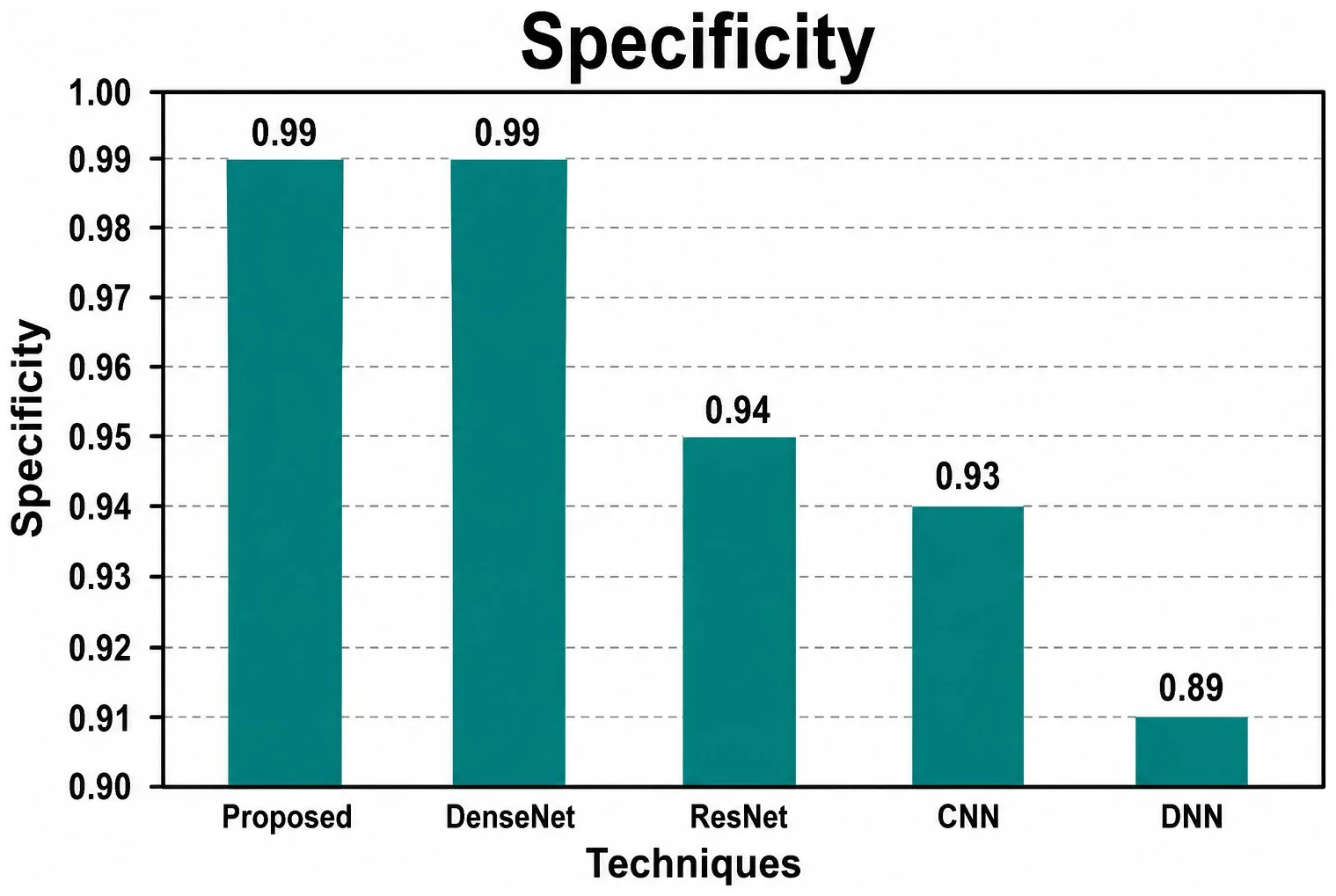
Specificity.

**Fig 8:**
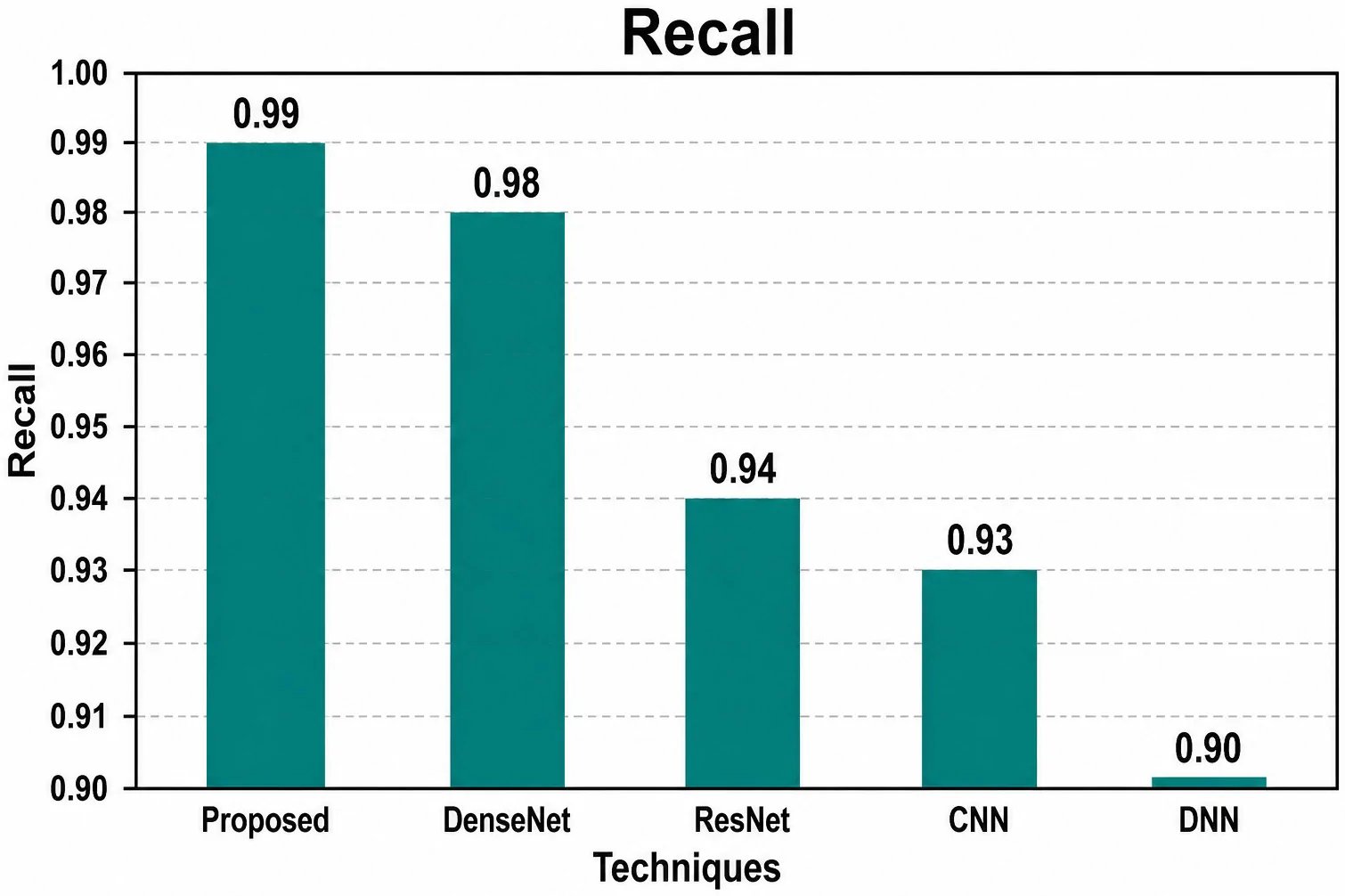
Recall.

**Fig 9:**
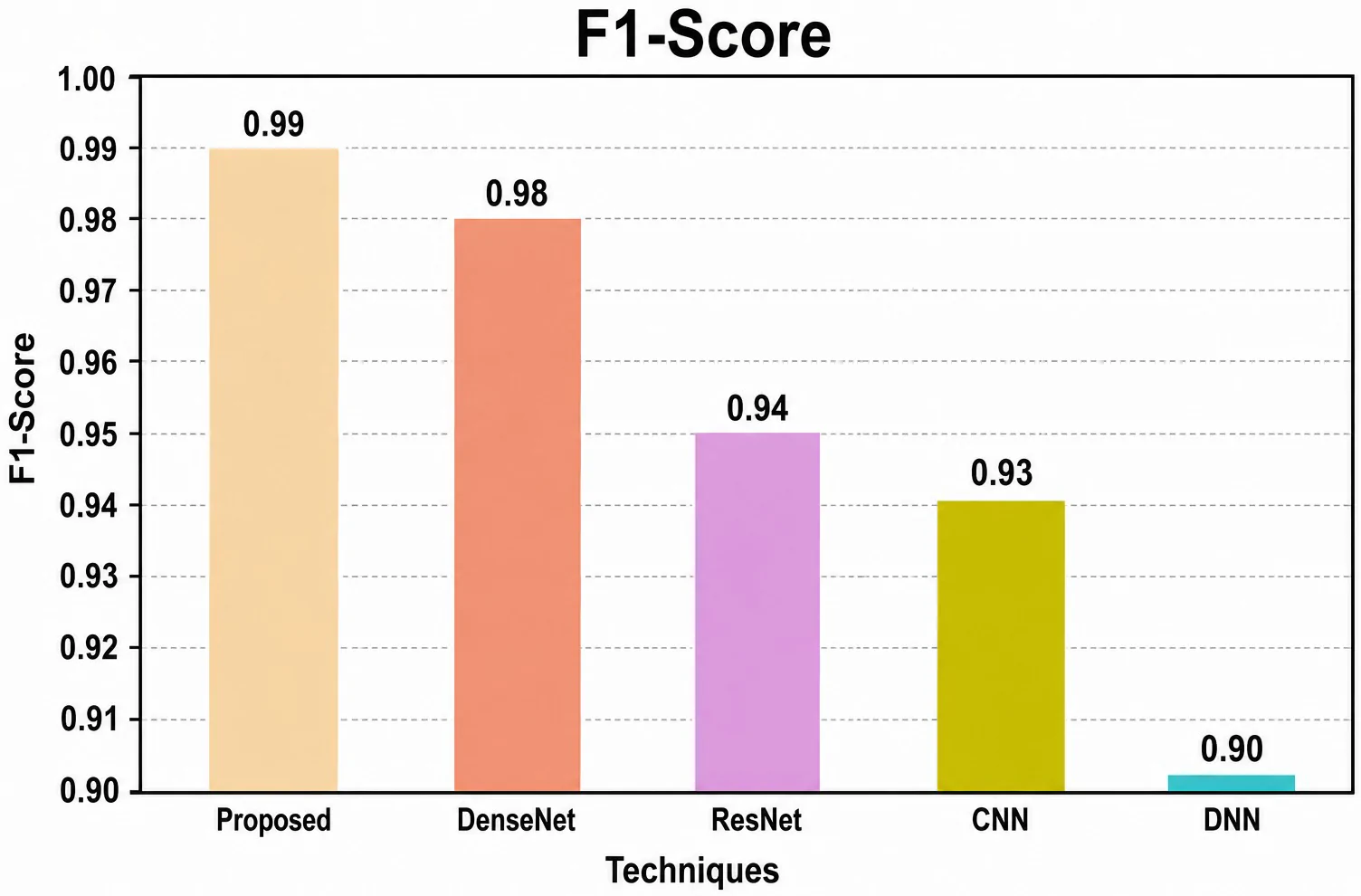
F1-Score.

**Fig 10:**
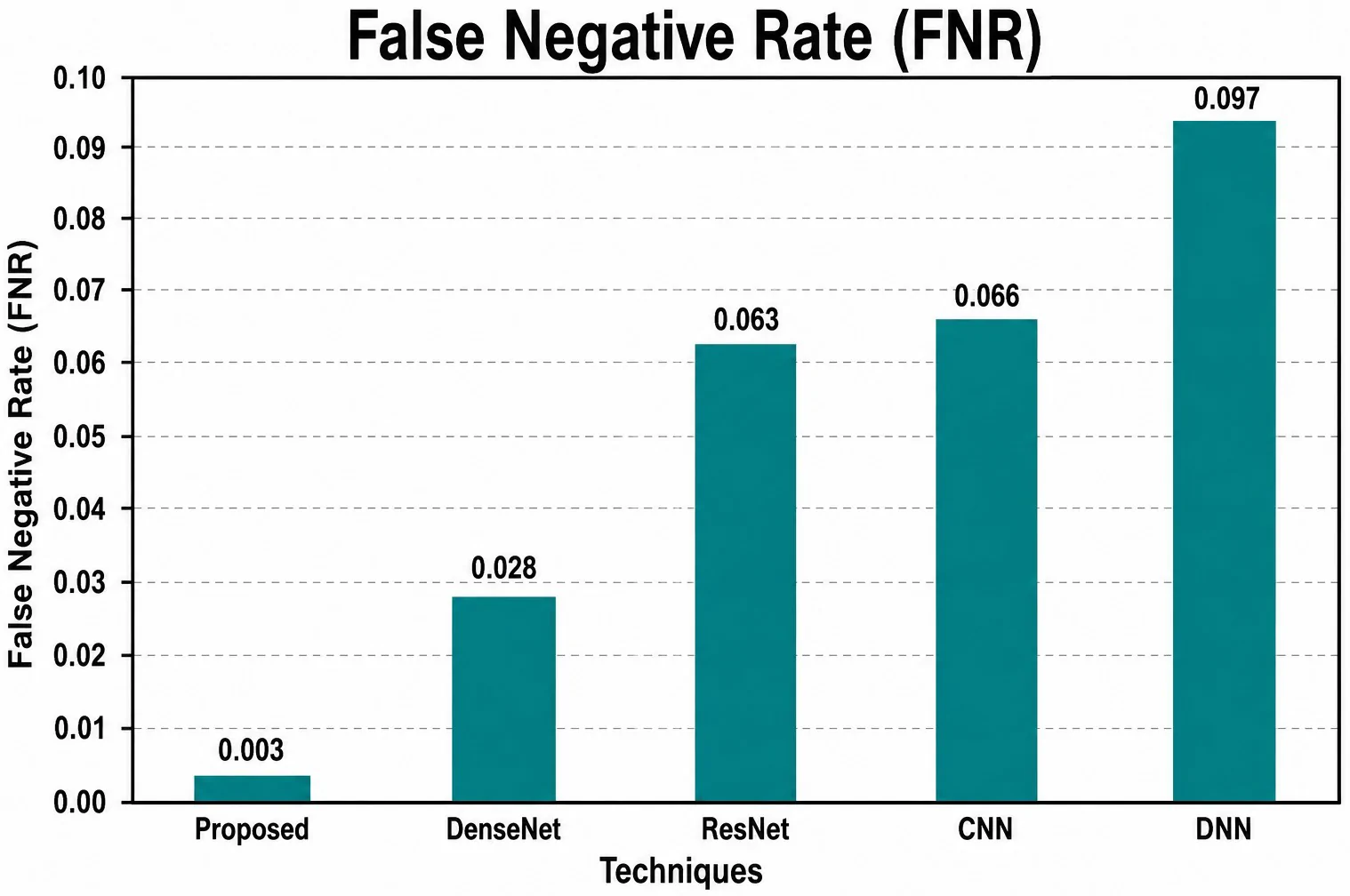
FNR.

**Fig 11:**
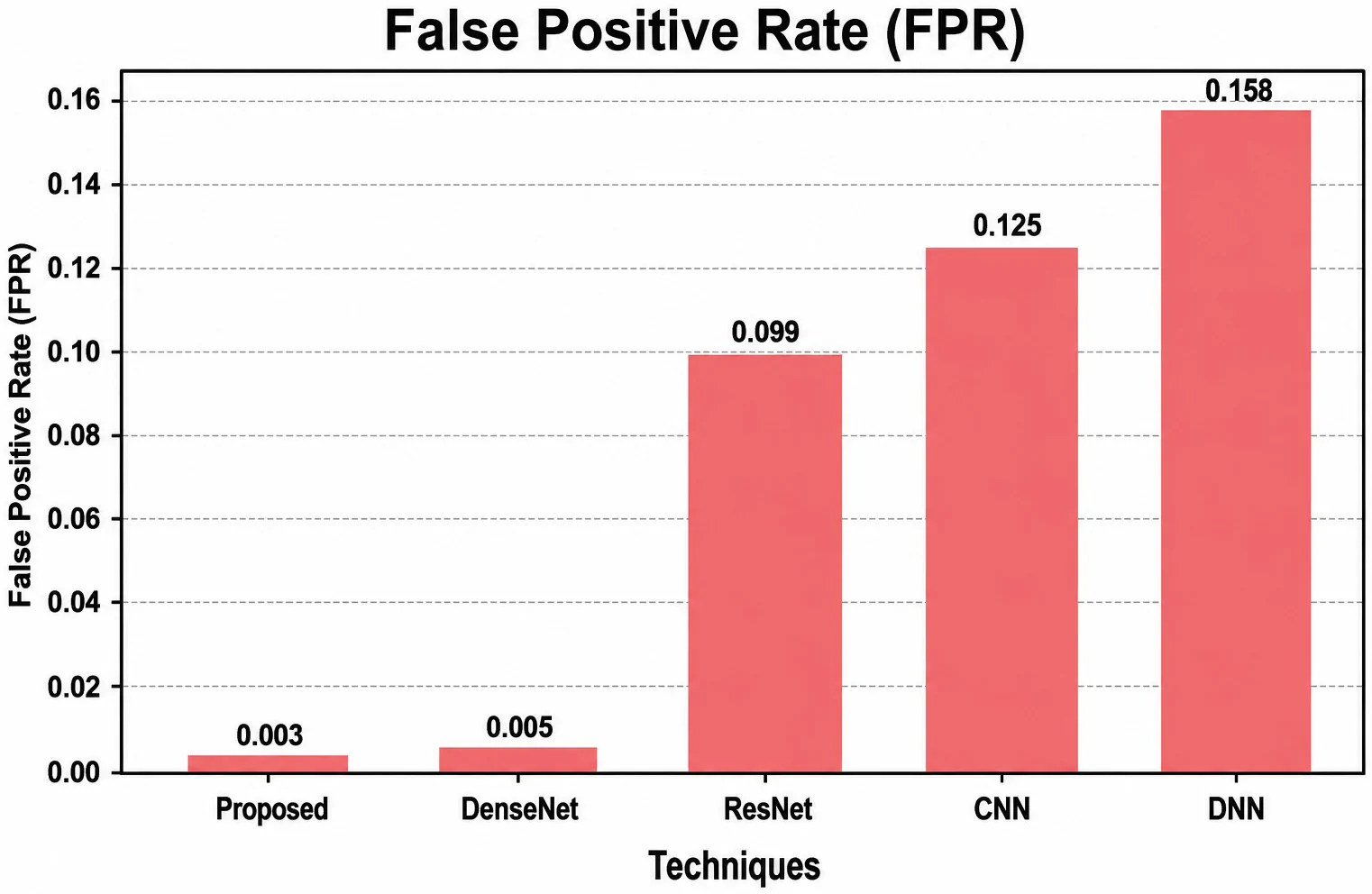
FPR.

The proposed model consistently outperforms the compared DenseNet, ResNet, CNN, and DNN approaches across the principal classification metrics, which can be observed from Fig 5. It achieves 99% accuracy, indicating a strong and balanced classification capability. DenseNet provides the closest competing performance, with 98% accuracy, while ResNet and CNN achieve 97% accuracy. The DNN exhibits the lowest overall performance among the evaluated methods.

The proposed model obtains the highest precision (Fig 6) of 0.99, followed by DenseNet (0.98), ResNet (0.97), CNN (0.95), and DNN (0.94). The higher precision of the proposed model indicates that a greater proportion of its positive predictions correspond to the correct animal category, thereby reducing incorrect positive classifications. Although DenseNet provides comparable performance with a precision of 0.98, the proposed model achieves a further improvement of 1 percentage point. The larger improvements over ResNet, CNN, and DNN demonstrate the stronger positive-prediction performance of the proposed approach.

The proposed model and DenseNet achieve the highest specificity of 99% (Fig 7), indicating a low rate of false-positive predictions. The proposed method nevertheless demonstrates an advantage over ResNet, CNN and DNN, whose specificity values are 94%, 93% and 89%, respectively.

The recall (Fig 8) comparison shows that the proposed model achieves the highest recall of 0.99, followed by DenseNet (0.98), ResNet (0.94), CNN (0.93), and DNN (0.90). The higher recall of the proposed approach indicates its ability to correctly identify a greater proportion of relevant animal instances. In comparison, the lower recall values obtained by CNN and DNN indicate a greater tendency to miss relevant instances.

The F1-score results (Fig 9) further demonstrate the balanced classification performance of the proposed model. The proposed approach achieves an F1-score OF 0.99, followed by DenseNet (0.98), ResNet (0.94), CNN (0.93), and DNN (0.90). Since the F1-score reflects the balance between precision and recall, the higher value obtained by the proposed model indicates a more favorable balance between correctly identifying relevant instances and minimizing incorrect positive predictions.

The false negative rate (FNR) results (Fig 10) demonstrate that the proposed model produces the lowest FNR of 0.003. DenseNet records an FNR of 0.028, whereas ResNet, CNN and DNN obtain 0.063, 0.066 and 0.097, respectively. Since a lower FNR indicates fewer relevant instances being incorrectly classified as negative, the results suggest that the proposed model is more effective in minimizing missed detections compared with the evaluated baseline approaches.

The False Positive Rate (FPR) comparison (Fig 11) shows that the proposed model achieves the lowest FPR of 0.003, closely followed by DenseNet at 0.005. ResNet, CNN and DNN exhibit considerably higher FPR values of 0.099, 0.125 and 0.158, respectively. The lower FPR indicates that the proposed approach generates fewer incorrect positive classifications, demonstrating improved discrimination between relevant and non-relevant instances.

Fig 12 presents the variation in training and testing loss with respect to the number of epochs. The training loss decreases consistently from approximately 0.70 at the beginning of training to 0.31 at the final epoch, while the testing loss decreases from approximately 0.80 to 0.37. The simultaneous reduction in both training and testing losses indicates that the model progressively improves its learning and predictive performance during training. Moreover, the testing loss does not exhibit an increasing trend while the training loss decreases during the later epochs, suggesting that no obvious evidence of severe overfitting is observed from the learning curves. The decreasing gap between the training and testing losses toward the final epochs further indicates relatively stable convergence of the proposed model.

**Fig 12:**
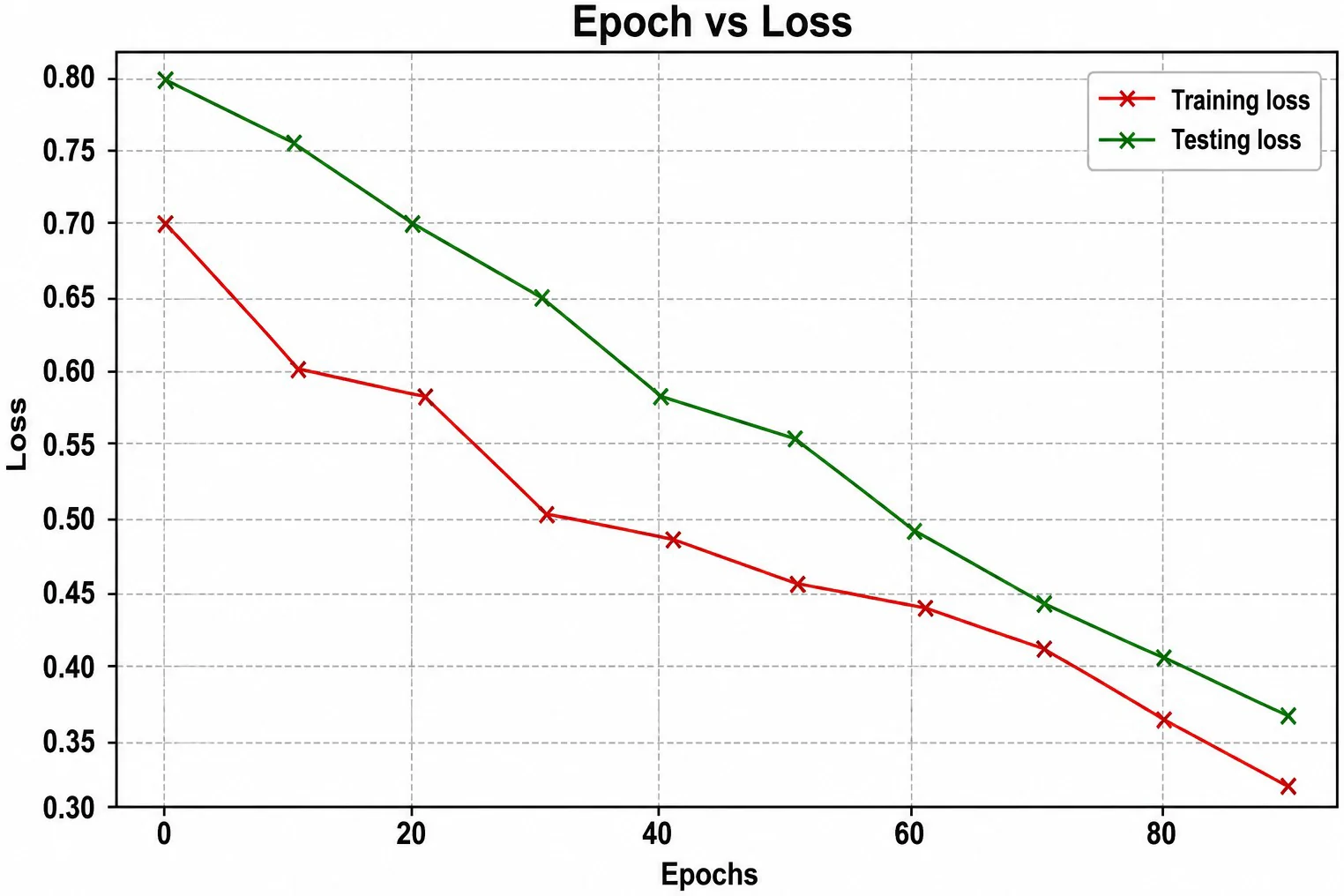
Loss analysis of OBSDLN method.

Fig 13 illustrates the variation in training and testing accuracy over the training epochs. Both training and testing accuracies exhibit an overall increasing trend as the number of epochs increases. The training accuracy improves from approximately 0.50 to 0.99, while the testing accuracy increases from approximately 0.32 to 0.97. The continuous improvement in testing accuracy indicates that the model progressively learns discriminative features and maintains its predictive capability on the testing data. Furthermore, no pronounced decline in testing accuracy is observed during the later epochs, while the training and testing accuracies remain relatively close toward the end of training. These observations suggest stable learning behaviour and provide no obvious evidence of severe overfitting from the accuracy learning curves.

**Fig 13:**
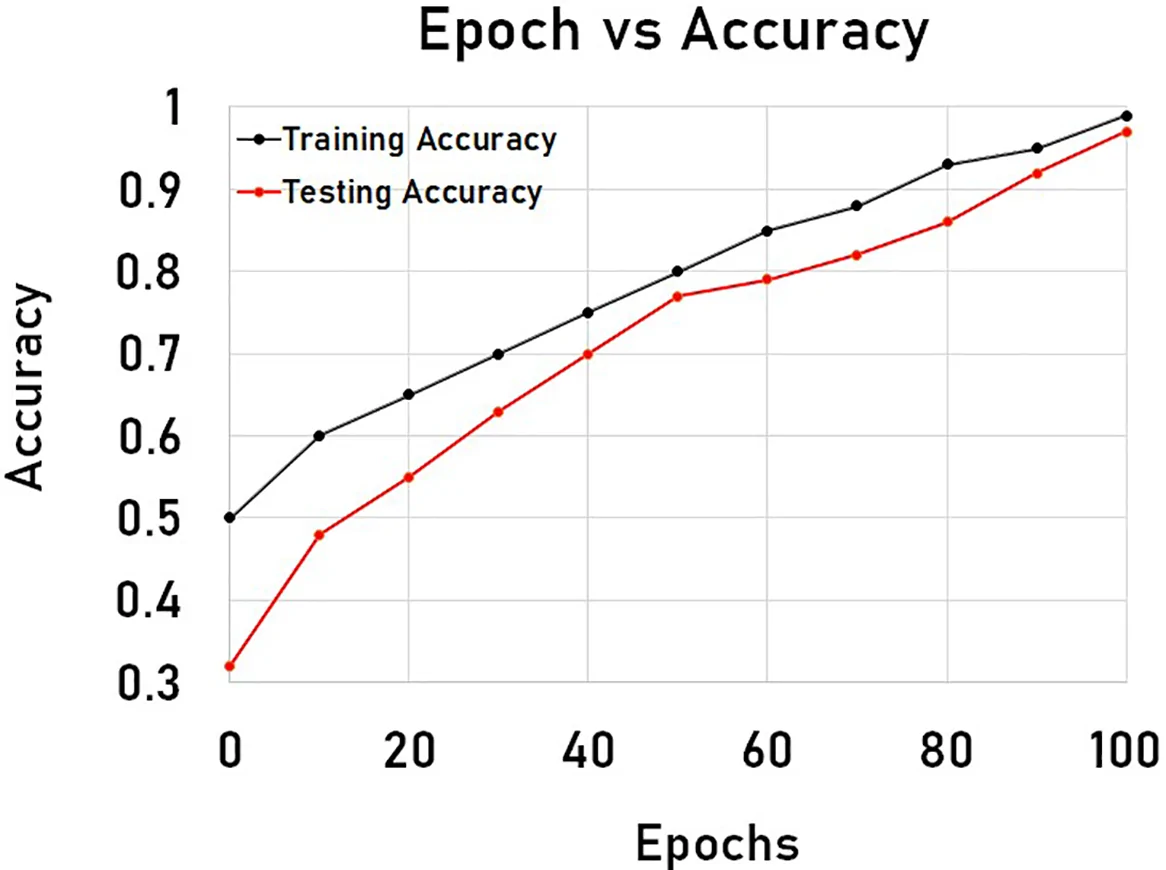
Accuracy analysis of OBSDLN method.

Fig 14 presents the ROC curve comparison of the proposed model with DenseNet, ResNet, CNN, and DNN. The roc curve illustrates the trade-off between the true positive rate and false positive rate at different classification thresholds. The proposed model maintains a higher true positive rate over a broad range of false positive rates and its curve lies closer to the upper-left region of the roc space compared with the competing approaches. DenseNet exhibits the closest performance to the proposed model, whereas ResNet, CNN, and DNN show comparatively lower true positive rates for corresponding false positive rates. These observations indicate that the proposed model provides stronger discriminative capability and can achieve a favorable balance between correctly identifying animal instances and limiting false-positive classifications.

**Fig 14:**
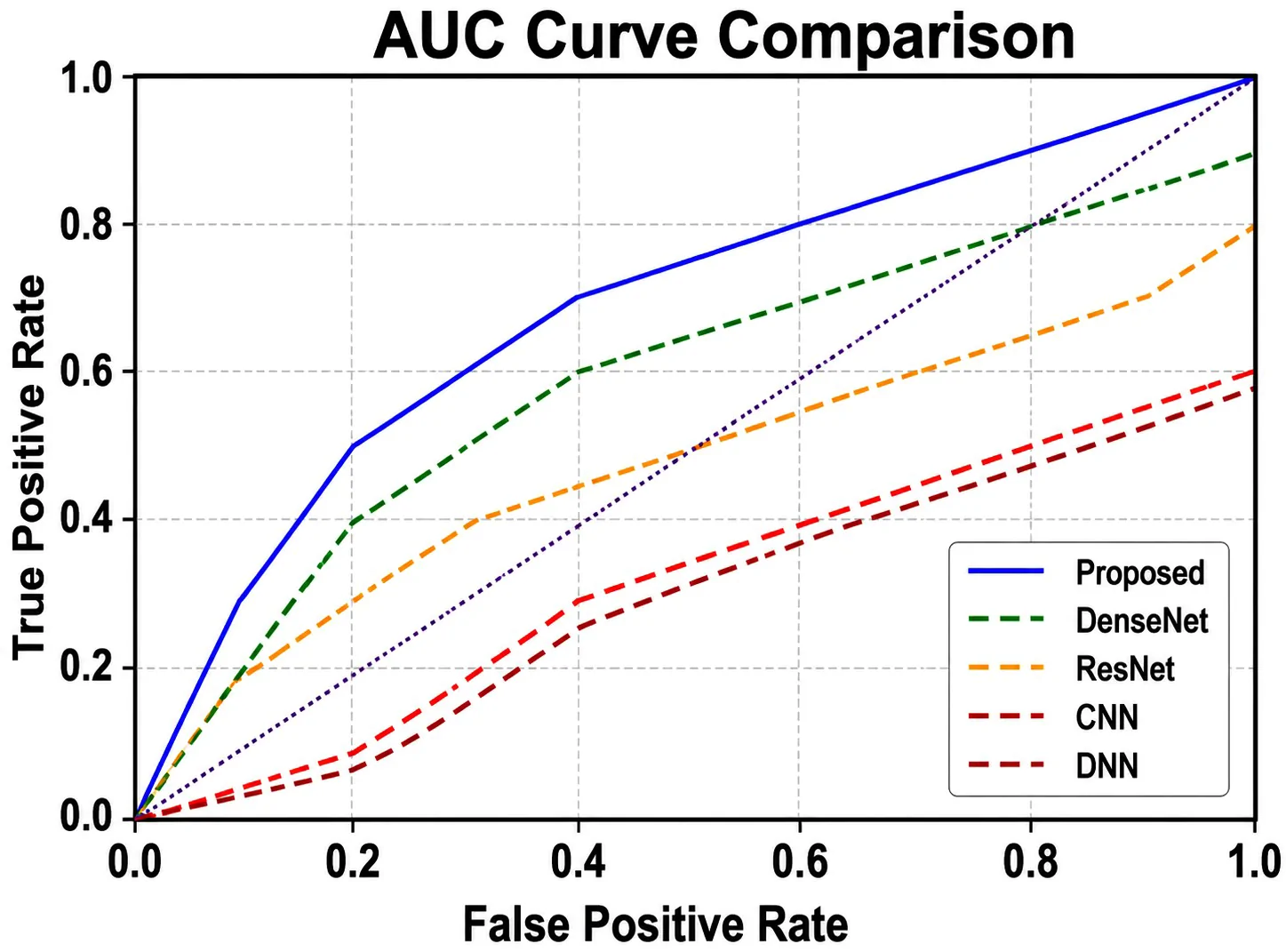
AU-ROC graph analysis.

Fig 15(a) compares the parameter complexity of the evaluated models. The proposed OBSDLN architecture contains approximately 1.7 million parameters, which is lower than DenseNet (7.0 million), ResNet (11.7 million), CNN (2.5 million), and DNN (2.1 million). The reduced parameter count indicates that the proposed architecture has a smaller model footprint while maintaining the required feature representation capability for animal identification. In particular, the proposed model contains approximately 85.5% fewer parameters than ResNet and 75.7% fewer parameters than DenseNet.

**Fig 15:**
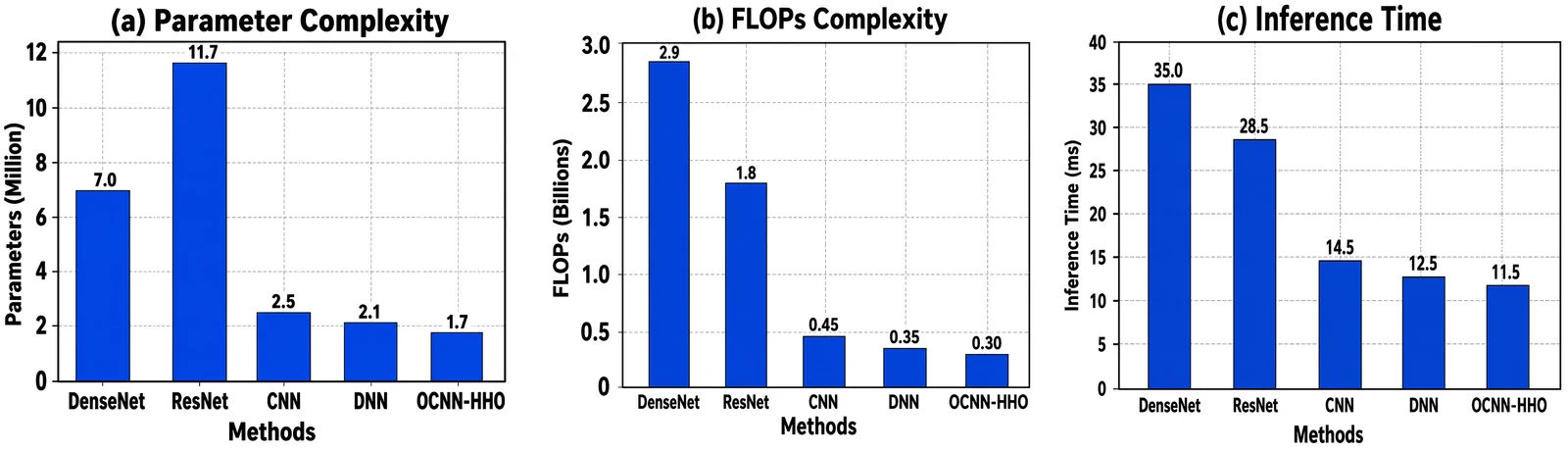
Computational complexity analysis.

The DenseNet confusion matrix (Fig 16(a)) demonstrates generally good classification performance, with class-wise correct prediction rates ranging from approximately 89% to 91%. However, several off-diagonal predictions of approximately 2–4% are observed across the classes, indicating that DenseNet has some difficulty distinguishing between visually similar animal categories. The ResNet confusion matrix (Fig 16(b)) shows improved class-wise classification compared with DenseNet. The correct prediction rates range from approximately 94% to 95% across the five classes, while the majority of misclassifications are limited to approximately 1–2%. This indicates that ResNet provides more consistent discrimination among the animal categories than DenseNet. The CNN confusion matrix (Fig 16(c)) indicates comparatively uneven class-wise performance. Class 3 achieves a particularly high correct classification rate of approximately 99%, whereas classes 1, 2, 4 and 5 achieve approximately 89%, 88%, 89% and90%, respectively. The presence of several 2–4% off-diagonal predictions for the latter classes suggests that the CNN experiences greater difficulty in distinguishing certain animal categories. The DNN confusion matrix (Fig 16(d)) exhibits the lowest and relatively uniform class-wise classification performance among the evaluated approaches, with correct prediction rates of approximately 86–87% across the five classes. Several off-diagonal predictions in the range of approximately 2–4% are also observed, indicating comparatively higher confusion between the animal categories. The confusion matrix of the proposed OBSDLN model (Fig 16(e)) demonstrates highly consistent classification performance across all five classes. Approximately 98% of the samples in each class are correctly classified, while the majority of the off-diagonal entries are limited to 0–1%. The uniform diagonal performance indicates that the proposed model does not exhibit a pronounced bias toward any particular class and is capable of effectively distinguishing the evaluated animal categories.

**Fig 16:**
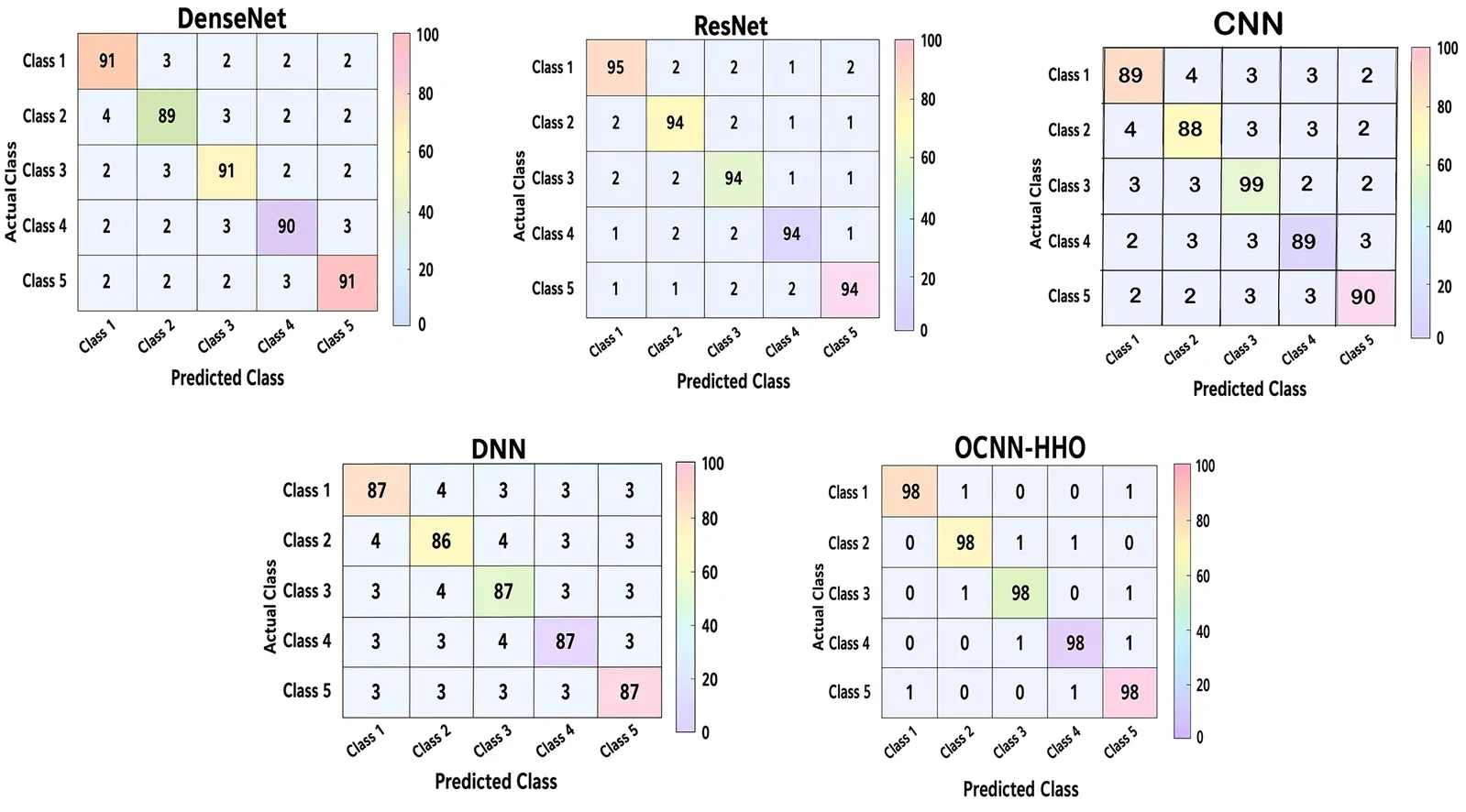
Confusion matrices of (A) DENSENET (B) RESNET (C) CNN (D) DNN and (E) proposed work (OBSDLN).

### Statistical significance analysis

To determine whether the observed performance differences among the evaluated models are statistically meaningful, a non-parametric statistical analysis was performed. The models were ranked according to their performance across the evaluation settings, with a lower average rank indicating better performance. A Friedman test was first used to assess whether significant differences existed among the compared techniques. The test indicated a statistically significant difference among the evaluated techniques (P < 0.05). Therefore, a Nemenyi post-hoc test was subsequently performed to identify the pairwise differences between the models (Fig 17).

**Fig 17:**
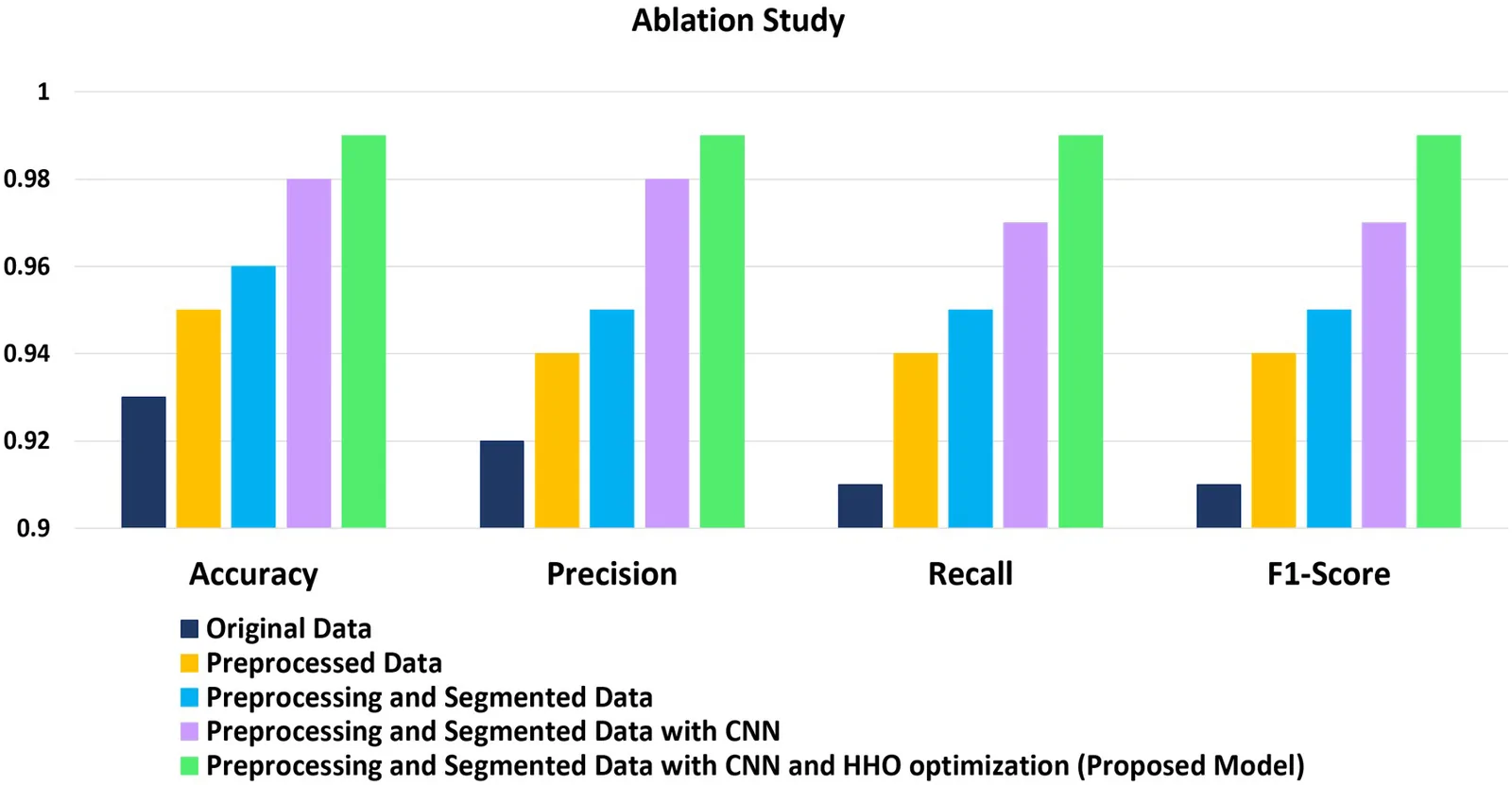
Ablation study analysis.

The resulting critical-difference diagram is presented in Fig 18. The proposed OBSDLN model obtained the lowest average rank (approximately 1.0), followed by ResNet, DenseNet, CNN and DNN. The critical difference was 1.639. The average-rank difference between OBSDLN and ResNet was smaller than the critical difference, indicating that their performance difference was not statistically significant. In contrast, the rank differences between OBSDLN and DenseNet, CNN and DNN exceeded the critical difference, indicating statistically significant differences for these comparisons. These findings provide statistical support for the superior performance of the proposed approach relative to most of the evaluated baseline models while avoiding an unsupported claim of significant superiority over ResNet (Fig 19).

**Fig 18:**
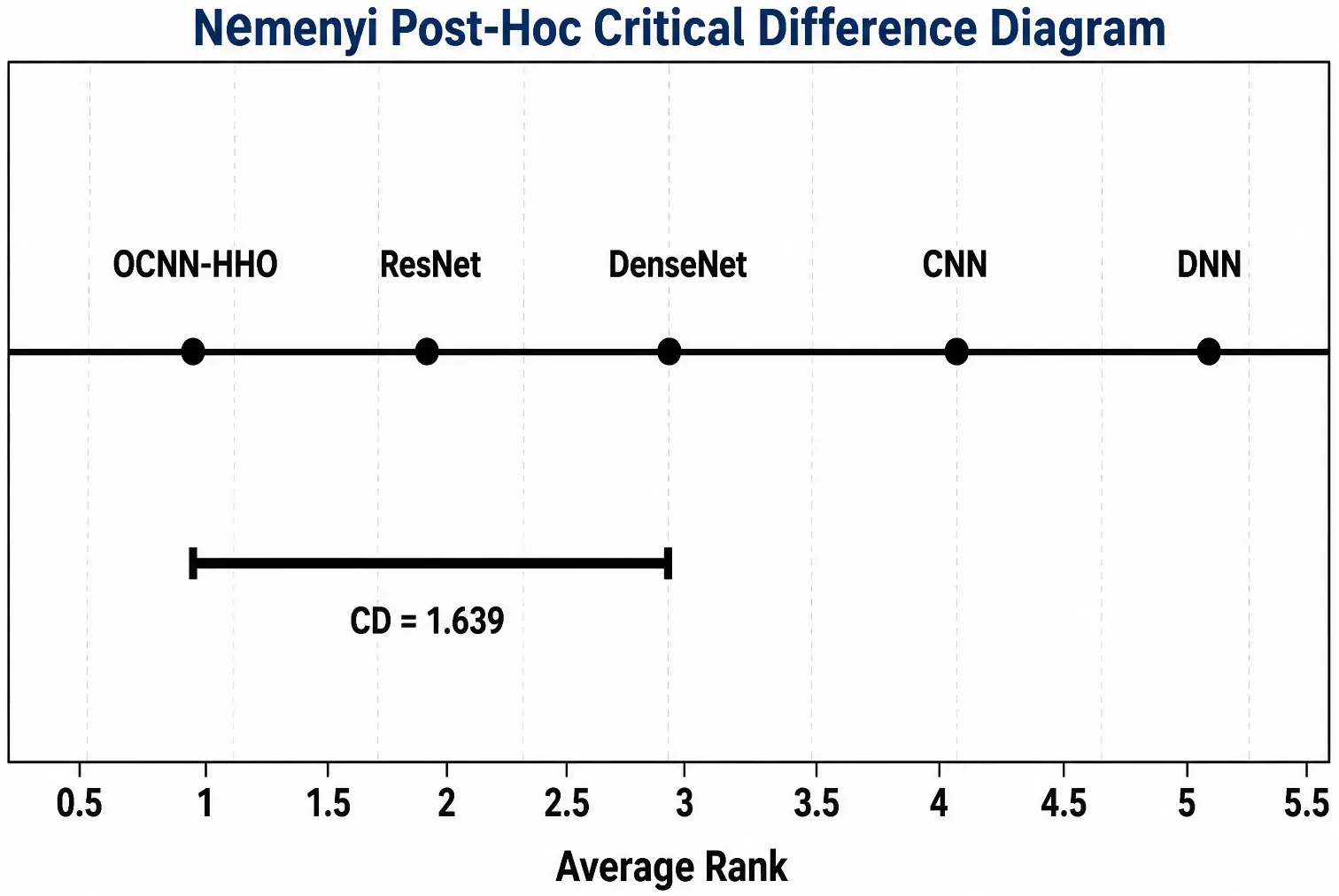
Nemenyi post hoc analysis.

**Fig 19:**
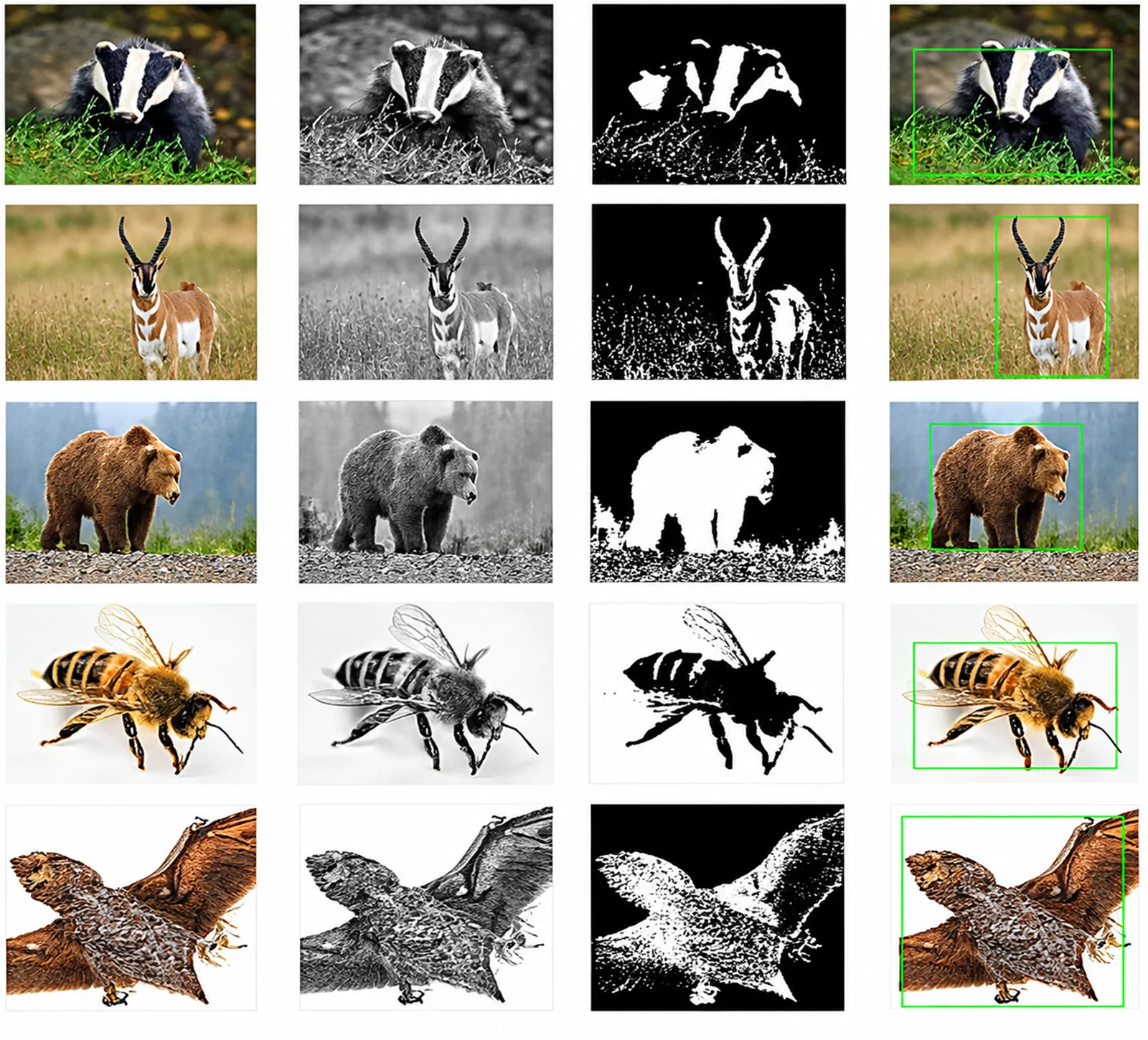
Output of the proposed method. Input images, pre-processed images, segmented images, predicted images.

The performance metrics of the proposed method are compared in Table 1. Where each performance metric is compared between the suggested approach and the existing approach. The results unequivocally demonstrate that the suggested performance metrics have the highest values. The accuracy of the suggested BSDLN is contrasted with earlier attempts in Table 2. The ablation study analysis with the suggested system is shown in Table 3. The results show that the recommended method achieves 99% accuracy when compared to the other novel research methodology. Because the suggested methodology made use of the BSDLN, it produced better results.

## Conclusion

In this research, OBSDLN method is used for wild animal detection. The open-source system database was used to evaluate the efficacy of the suggested methodology. Four phases that make up the suggested approach are data collection, pre-processing, segmentation and detection. Initially, input image is pre-processed using a Median filter and AHE. After that, BSDLN will be used to split the pre-processed images into segments that correspond to the wild animal. After segmentation process, segmented output is sent to CNN for wild animal detection. Ultimately, the HHO is utilized to adjust the weight variable of the CNN model that has been trained to detect wild animals. The effectiveness of the recommended strategy is assessed using specific performance standards, such as accuracy, F1-measure, recall, precision, and specificity. The accuracy of the recommended approach is 99%. Our objective is to enhance the research for next projects by applying DL methods and more sophisticated datasets to the identification of untamed creatures. The dataset can be expanded in further research to diversify the findings Several backdrop pictures with similar designs might be utilized in order to minimize false detection.
